# Closing the Polyurethane Loop: Repolyols from Chemically Recycled Bio-Modified Foams for Viscoelastic Applications

**DOI:** 10.3390/polym18172142

**Published:** 2026-09-02

**Authors:** Michał Kucała, Elżbieta Malewska, Maria Kurańska, Aleksander Prociak

**Affiliations:** 1 Department of Chemistry and Technology of Polymers, Faculty of Chemical Engineering and Technology, CUT Doctoral School, Cracow University of Technology, Warszawska 24, 31-155 Cracow, Poland; michal.kucala@doktorant.pk.edu.pl; 2 Department of Chemistry and Technology of Polymers, Faculty of Chemical Engineering and Technology, Cracow University of Technology, Warszawska 24, 31-155 Cracow, Poland; 3 Interdisciplinary Centre for Circular Economy, Cracow University of Technology, Warszawska 24, 31-155 Cracow, Poland

**Keywords:** viscoelastic polyurethane foams, flexible foams, Circular Economy, bio-foams, repolyols

## Abstract

This article develops new-generation viscoelastic polyurethane foams (VPUFs) obtained using repolyols derived from chemically recycled flexible foams and bio-based polyurethane foams, in accordance with the Circular Economy framework. The repolyols were produced by chemolysis using a low-molecular-weight glycolyzing agent. The repolyols were used as a partial substitute for conventional petrochemical polyols in VPUF formulations. The physicochemical properties of the repolyols, including hydroxyl value (HV), amine value (AmV), and viscosity, were characterized. The effect of the repolyols on the foaming reaction and the cellular structure of the resulting foams was assessed. The resulting VPUFs were tested for apparent density, hardness, hysteresis, resilience, recovery time, and comfort factor. Replacing up to 15% by weight of conventional petrochemical polyols with repolyols results in VPUF with 36% higher apparent density, 32% higher hardness and 27% higher hysteresis than REF. The developed polyurethane foams are in line with the principles of sustainable development and the Circular Economy.

## 1. Introduction

Among flexible polyurethane foams (FPUFs), viscoelastic polyurethane foams (VPUFs) are characterized by specific physical and mechanical properties such as apparent density, hysteresis and resilience [1]. Similarly to FPUFs, appropriate modification of the raw materials used allows for a wide range of hardness and other functional properties of VPUFs [2]. VPUFs soften at a moderate temperature increase [3] and maintain dimensional stability under their own weight at temperatures below 90 °C, which allows for their use in elevated temperature conditions, provided there are no significant mechanical loads [4]. Unlike rigid polyurethane foams (SPUF), which reach their elastic limit at approximately 10% deformation, VPUFs can undergo deformations of up to 90%, while maintaining the ability to regain their original shape after the load is removed (the change in linear dimensions does not exceed 10%) [1]. VPUFs are usually characterized by an open-cell structure, which favours their ability to absorb water [5]. Foams are also capable of absorbing moisture present in the air [6]. The presence of the open-cell structure of foams also contributes to their favourable acoustic insulation properties [7]. VPUFs have a very high ability to dampen vibrations, impacts, and shocks [8]. Due to these properties, VPUFs are used, among other products, in the production of dashboards and packaging materials [4]. VPUFs are obtained in a single-stage process [9] using toluene diisocyanate (TDI), methylene diphenyl diisocyanate (MDI) [10], or components with a high content of free isocyanate groups, which have properties similar to conventional isocyanate raw materials. VPUFs are usually obtained with a low isocyanate index (I_NCO_), which favours the presence of numerous free polymer chains and incomplete cross-linking of the foam structure [9]. The viscoelastic properties and the deformation mechanism are determined by the chemical structure of the polyols used. Some polyols used to obtain VPUFs are characterized by a high hydroxyl value (HV), approximately 400 mgKOH/g and functionality ≥ 2, which leads to shortening of the polymer chain segments between the network nodes and consequently affects the strength of the foams. To increase the flexibility of the material, additional polyols with a low HV (approximately 30 mgKOH/g) are introduced into the reaction system [3]. The ability of VPUFs to gradually recover their original shape after the removal of the deformation stress is due to their phase structure [1].

Until the beginning of the second decade of the 21st century [11], most polyols were produced from products derived from the petrochemical industry. The need to find alternative sources of raw materials, particularly in the face of growing environmental problems, has fuelled the development of biopolyols. They are most often obtained from vegetable oils, such as rapeseed [12], sunflower [13], corn [14], linseed [15], soybean [16], palm [17], coconut [18], or peanut [19], using various synthesis methods. Biopolyols can also be produced from used frying oils [20]. They are mainly used as an alternative raw material in the production of polyurethanes [21]. Although a given biopolyol is derived from the same plant source and has similar applications, its physicochemical properties may differ significantly depending on the synthesis method used. Biopolyols are usually colourless or yellowish-brown liquids [22,23,24]. They vary in viscosity, which depends on the average molecular weight and the content of hydroxyl groups—increasing these properties leads to an increase in the viscosity of biopolyols [25]. Due to the similarity of chemical properties to the vegetable oils from which they are obtained, biopolyols are prone to rancidity [24]. This process involves autoxidation of bonds in the fatty acid chain, which leads to the formation of low-molecular-weight odorous compounds such as aldehydes, ketones, and carboxylic acids [26].

Chemical recycling leads to partial degradation of polyurethane materials, resulting in the formation of oligomeric fractions with a low average molecular weight and low-molecular-weight compounds [27]. Chemolysis processes involve mono- or bifunctional reagents with a low molecular weight [28]. The obtained products can be reused as raw materials (repolyols) or other components in the synthesis of both the same and different materials [29]. All chemolysis methods lead to the cleavage of selected bonds in the polyurethane structure, especially urethane, urea, allophanate, and biuret bonds [27]. The increasing scale of polyurethane foam production [30] is associated with the generation of significant amounts of both post-production and post-consumer waste. Due to their low apparent density, these materials occupy a large volume during storage. In the context of the Circular Economy [31], actions are being taken to reduce their addition to landfill. Alternative methods of polyurethane waste management include mechanical and chemical recycling, raw material recovery, and energy recovery [32,33,34]. The most used method of chemical recycling of polyurethanes is glycolysis [27,35,36]. It involves the degradation of the polyurethane material under controlled conditions, using glycols or their mixtures with amines, leading mainly to the cleavage of urethane and/or urea bonds [27]. The process usually uses low-molecular-weight glycols containing up to six carbon atoms, such as ethylene glycol [37], propylene glycol [37], butylene glycol [38], diethylene glycol (DEG) [39] or dipropylene glycol [40], as well as polyether glycols, e.g., polyethylene and polypropylene glycol [41]. The catalysts used include, among others, hydroxides (potassium [42], sodium [40]), acetates (potassium [36], sodium [43], zinc [44]) and tin compounds, such as tin (II) octanoate [37] or dibutyltin (IV) dilaurate [45]. The type of reagents used determines the process parameters, including temperature (170–220 °C) and duration (15–360 min), as well as the physicochemical properties of the obtained products, i.e., repolyols, which contain terminal hydroxyl and/or amine groups [29,46,47,48,49,50].

Although the synthesis of FPUFs modified with biopolyols and the glycolysis of conventional polyurethane foams have been described, the potential for chemical recycling of FPUFs containing biopolyols and the use of the resulting rebiopolyols for the synthesis of new-generation viscoelastic foams has not been explored to date. This article describes for the first time the glycolysis of FPUFs modified with biopolyol derived from rapeseed oil and assesses the potential for using the resulting repolyol as a partial substitute for petrochemical polyols in the synthesis of VPUFs. The effects of the type and content of repolyol on the foaming process, cellular structure, and physicochemical and mechanical properties of the resulting foams were assessed.

## 2. Materials and Methods

### 2.1. Materials

Biopolyol (B) was obtained by epoxidation of unsaturated bonds in higher fatty acid residues of rapeseed oil, followed by opening of the oxirane rings of the resulting epoxy derivatives. Epoxidation of rapeseed oil (Olej Kujawski, Bunge Polska Sp. z o.o., Kruszwica, Poland) was carried out using peracetic acid obtained in situ by mixing 99.5% acetic acid (Avantor Performance Materials Polska S. A., Gliwice, Poland) with 30% hydrogen peroxide solution (Chempur, Piekary Slaskie, Poland) in the presence of Amberlite^®^ IRC120 H ion exchange resin in the hydrogen form (Sigma-Aldrich, Saint Louis, MO, USA). Subsequently, the oxirane rings of the epoxy derivatives of the oil were opened using diethylene glycol—DEG (Chempur, Piekary Slaskie, Poland) and sulfuric acid (VI) catalyst (Avantor Performance Materials Polska S. A., Gliwice, Poland).

The petrochemical polyol Rokopol^®^ F3600 (PCC Group, Brzeg Dolny, Poland) was used to prepare FPUFs of different types: without (FP), or with the biopolyol (FB). In the EPPU FB formulations, biopolyol B was used as a substitute for 20% by weight of the petrochemical polyol. Two catalysts were used: the organometallic Dabco^®^ T-9 (Evonik Industries AG, Essen, Germany), and the amine Dabco^®^ BLV (Evonik Industries AG, Essen, Germany). Vorasurf™ DC 5906 (Dow Inc., Midland, MI, USA) was used as a surfactant, and demineralized water was used as a blowing agent. TDI Lupranat^®^ T 80 M (BASF SE, Ludwigshafen am Rhein, Germany) was used the isocyanate component.

Both foam types FP and FB were chemically recycled. Table 1 presents the FPUF formulations subjected to glycolysis. The resulting repolyols were marked as rFP and rFB. In the chemical recycling of FPUF, DEG was used as a chemolyzing agent, while potassium hydroxide (Avantor Performance Materials Polska S. A., Gliwice, Poland) in the form of flakes was applied as a catalyst.

In the synthesis of VPUF (V_rFP, V_rFB), the petrochemical polyol mixture of Rokopol^®^ RF551, Rokopol^®^ M6000 and Rokopol^®^ M1170 (PCC Group, Brzeg Dolny, Poland) was used. The repolyols rFP and rFB were used as replacements of petrochemical polyols. Dabco^®^ DC 2 (Evonik Industries AG, Essen, Germany) was used as a catalyst, Niax^TM^ Silicone L-627 (Momentive Inc., Niskayuna, NY, USA) as a surfactant, and demineralized water as a chemical blowing agent. PMDI Suprasec^®^ 2424 (Huntsman Corporation, The Woodlands, TX, USA) was used as the isocyanate component.

### 2.2. Methods

#### 2.2.1. Synthesis of Biopolyol

The biopolyol was obtained by epoxidation of unsaturated bonds in rapeseed oil and opening of the resulting oxirane rings. The epoxidation reaction was carried out in a glass reaction vessel equipped with a mechanical stirrer and a heating jacket filled with circulating technical DEG (Brenntag Polska Sp. z o.o., Kędzierzyn-Koźle, Poland). The temperature of the technical DEG was regulated by a Huber Compatible Control CC 308 device (Peter Hyber Kältemaschinenbau SE, Offenburg, Germany). The biopolyol was obtained by epoxidation carried out in a reaction vessel filled with ion exchange resin. Rapeseed oil was added to the reaction vessel and mixed with 99.5% acetic acid. The mixture was heated to 60 °C, and then 30% hydrogen peroxide was added portion-wise. The reaction temperature was maintained at a level not exceeding 65 °C. The reaction was carried out for 2.5 h until the epoxide value (LEP) reached 0.14 mol/100 g of epoxidized rapeseed oil.

The epoxidized rapeseed oil was poured into separatory funnels, where the oil phase was separated from the acidic aqueous phase. The residual acid was poured from the funnels, and the oily phase was washed with hot tap water until the decanted aqueous phase reached a pH of ≈7.

To remove residual water from the neutralized, epoxidized rapeseed oil, vacuum distillation was performed using an Ilmvac 105 T-10 ef system (Ilmvac GmbH, Ilmenau, Germany). The neutralized oil was transferred from the separatory funnels to a round-bottom flask, which was then connected to a vacuum distillation set-up. The mixture was heated in a heating mantle to 55 °C, and then the system pressure was gradually reduced to approximately 15 mbar. The distillation process was terminated when the distilled epoxy oil became transparent, and no more water condensed from the condenser.

Distilled epoxy derivatives of rapeseed oil were transferred to a 6-L glass reaction vessel equipped with a mechanical stirrer and a heating mantle. Based on the oxirane ring content, the mass of DEG required to open them was determined, assuming the number of moles of epoxy groups corresponds to the number of moles of DEG used. The mass fraction of the catalyst during the ring-opening reaction was 0.3% of the mass of epoxidized rapeseed oil. Concentrated sulfuric acid (VI) was used as the catalyst. The acid catalyst was premixed with DEG before addition to the reaction vessel to limit local degradation of the epoxidized vegetable oil. The oxirane ring-opening process was conducted at 90 °C until an epoxy value of no more than 0.01 mol/100 g of biopolyol was obtained.

#### 2.2.2. Synthesis of Repolyols

The FPUF was cut into pieces on a band saw and then ground in a mechanical mill to obtain foam flakes.

Chemical recycling of the foams was carried out in a glass reaction vessel equipped with a mechanical stirrer and a heating mantle. The glycolysis agent (DEG) and catalyst (KOH) were initially introduced into the reaction vessel. The mass fraction of glycolyzed foam to DEG was 1.5:1, while the catalyst content was 1% by weight relative to the amount of DEG used. The DEG–catalyst mixture was heated to 180 °C at a stirring speed of 200 rpm.

Once the desired reaction temperature was reached, the stirrer speed was increased to 250 rpm and the addition of ground foam began. The foam was added in 50 g portions every 15 min. After the last portion of foam was added, the stirrer speed was increased to 350 rpm and the reaction mixture was maintained at the reaction temperature for another 2 h. This resulted in the production of repolyol.

The glycolysis reaction of FPUF yielded repolyols (recyclates). Two FPUFs were subjected to glycolysis, yielding the following recyclates:

- rFP—recyclate of FPUF unmodified with biopolyol (reference);
- rFB—recyclate of FPUF in which 20% by weight of the petrochemical polyol was replaced with a biopolyol with an LOH of 153 mgKOH/g.

The formulations of which are presented in Table 1.

The repolyols were not purified and were used in VPUF formulations as a homogenized mixture.

#### 2.2.3. Characteristics of Biopolyol and Repolyols

The physicochemical characterization of biopolyol and repolyols was performed by determining the following: HV (ISO-4629-2 [51]), acid value (PN-EN ISO 2114 [52]), AmV (BN-69/6110-29 [53]), epoxide value (ASTM D1652-11 [54]), density (PN-EN ISO 2811-1 [55]), and percentage water content by the Karl Fischer method (PN-81/C-04959 [56]). Viscosity was measured using an RM 200 Plus CP-4000 Plus rotational rheometer (Lamy Rheology Instruments, Champagne au Mont d’Or, France). Chromatographic analysis was also performed using a Knauer chromatograph (Knauer Wissenschftliche Geräte GmbH, Berlin, Germany). The chromatographic system included a precolumn and an AppliChrom^®^ column (AppliChrom GmbH^®^, Oranienburg, Germany) with a porosity of 35 Å and a molar mass range from 100 to 2500 g/mol. The column bed consisted of a styrene-divinylbenzene copolymer with a particle diameter of 5 μm. The instrument was calibrated using poly(methyl methacrylate) standards. Stabilized tetrahydrofuran was used as the mobile phase, fed at a flow rate of 0.5 cm^3^/min. Measurements were performed at 35 °C. GPC analysis allowed the determination of number (Mn) and mass (Mw) average molecular weight, molecular weight dispersity (d), functionality (f), and percentage DEG content, which was determined based on the percentage area of the DEG peak in the chromatogram. FTIR analysis was performed using a Nicolet™ iSTM 5 spectrophotometer (Thermo Fischer Scientific Inc., Waltham, MA, USA).

#### 2.2.4. Flexible and Viscoelastic Polyurethane Foams

FPUF and VPUF were obtained by a single-step method, according to the calculated recipes. FPUF was modified with biopolyol B, replacing the petrochemical polyol in the foam recipe by 20% by weight. The flexible foam recipes—the reference one and the one in which the petrochemical polyol was replaced by biopolyol B by 20% by weight—are presented in Table 1.

VPUFs modified with FPUF rFP and rFB repolyols were obtained by partial replacement of petrochemical polyols with 5% wt, 10% wt, 15% wt, and 20% wt of recyclates. The compositions of V_rFP and V_rFB formulations, in which the share of repolyols did not exceed 20% wt, are summarized in Table 2 and Table 3.

Non-reactive ingredients were weighed on a laboratory scale, i.e., polyols, biopolyol, repolyols, catalysts, chemical blowing agent—demineralized water and surfactant. Then the obtained reaction mixture was stirred for about 1 min using a mechanical mixer, obtaining the so-called polyol masterbatch.

The isocyanates were weighed on a laboratory scale in a separate container and then vigorously poured into the container containing the polyol premix. The resulting polyurethane composition was then mixed for approximately 5 s using a mechanical stirrer and poured into a polypropylene mold, where the foam material was allowed to rise freely.

After the growth process was completed, the foams in molds were seasoned for 1 h at 70 °C and left for further seasoning for 23 h at ambient temperature. After seasoning, the foams were removed from the molds and samples were cut out using a band saw.

Three compositions from each polyurethane system were obtained and subjected to foaming process analysis. Three foams from each polyurethane system were poured and tested for their chemical and cellular structure as well as their physicomechanical properties.

The foaming process of polyurethane compositions was analysed using the FOAMAT 285 Universal Foam Qualification System (Format Masstechnic GmbH, Karlsruhe, Germany). The study determined characteristic foaming times, maximum foam core temperature, maximum intra-pore pressure, shrinkage ratio, maximum foam growth rate, and dielectric polarization. Cell structure analysis was performed using SEM images of the foams taken using an SEM TM3000 scanning electron microscope (Hitachi, Tokyo, Japan). SEM images were interpreted using Fiji ImageJ analytical software (ImageJ 1.54p, U.S. National Institutes of Health, Bethesda, MD, USA). The number of cells per mm^2^ of the SEM image, cell cross-sectional area, anisotropy coefficient, and cell density were determined. Three images of selected foam from each polyurethane system were taken. The images were taken parallel to the foam growth direction in different areas of the material. The open cell content was tested according to ISO 4590 [57]. The content of flexible segments (FS) and rigid segments (RS) was calculated according to the formula described in the article by Kucała et al. [42]. The apparent density of the foams was determined according to ISO 845 [58]. The hardness (ISO 3386-1 [59]), hysteresis (ISO 2439 [60]), and comfort factor of the foam materials were tested using a Zwick/Roell Z005 TH Allround-Line testing machine (Zwick Roell Group, Ulm, Germany). The recovery time of the foams was determined according to the IKEA^®^ specification IOS-MAT-0076 [61], and the rebound was determined according to ISO 8307 [62]. All measurements were performed in triplicate, and the results are presented as mean values with standard deviations (SD).

### 2.3. Graphical Abstract Preparation

The graphical abstract was generated using the Illustrae platform (https://illustrae.co/ accessed on 15 July 2026) based on the content of the manuscript, with the abstract of the manuscript used as the prompt/input. As Illustrae is an online platform, no specific software version is provided to users. The generated graphical content was subsequently reviewed and adapted by the authors to ensure its scientific accuracy and consistency with the scope and findings of the study. The authors take full responsibility for the final content of the graphical abstract.

## 3. Results and Discussion

**Figure 1:**
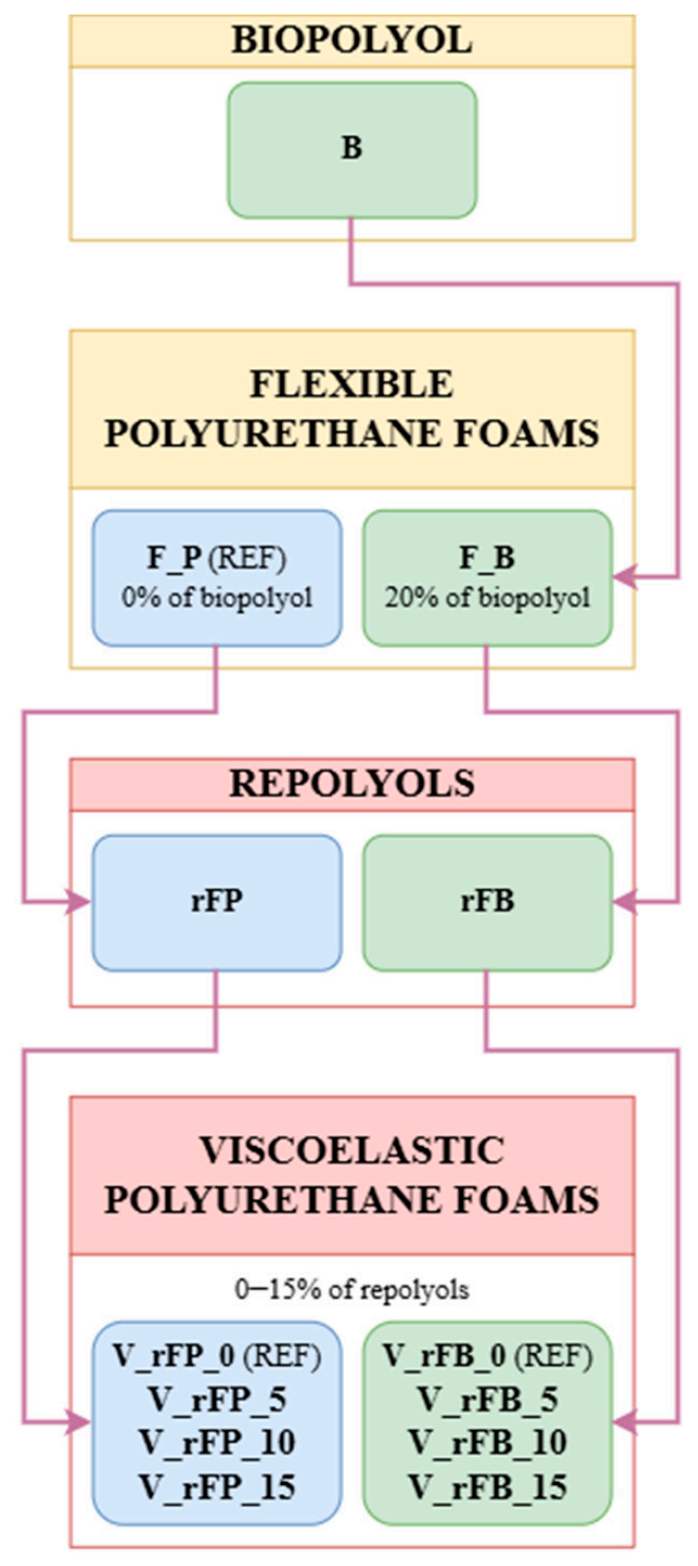
Conceptual diagram of the sequence of conducted research.

Figure 1 presents the concept and sequence of the research described in this article. First, biopolyol B was synthesized, which replaced 20% by weight of the petrochemical polyol in the formulation of flexible bio-polyurethane foam. A petrochemical FPUF F_P was also obtained as a reference material. The flexible foam and bio-polyurethane foam were chemically recycled to obtain repolyols—rFP and rFB, respectively. The repolyols were used as modifiers of the VPUF formulations, replacing petrochemical polyols in their formulations by 5% by weight (V_rFP_5, V_rFB_5), 10% by weight (V_rFP_10, V_rFB_10), and 15% by weight (V_rFP_15, V_rFB_15). An unmodified VPUF was also obtained as a reference material (V_rFP_0, V_rFB_0).

### 3.1. Biopolyol

The obtained biopolyol was characterized and its physicochemical properties are presented in Table 4.

**Figure 2:**
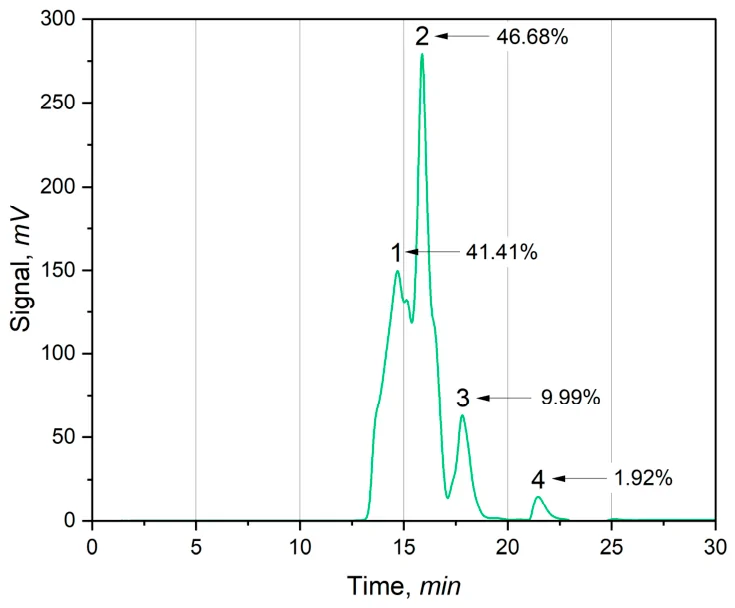
GPC chromatogram of biopolyol B.

In the chromatogram of the biopolyol B, three distinct peaks (1–3) are observed located in a relatively narrow retention time range of 13 to 18 min (Figure 2). Additionally, a weak signal (signal 4) is visible at a retention time of approximately 22 min, which can be attributed to the lighter fractions of molecular chains originating from unreacted DEG. The graph shows the percentages of the surface areas of individual peaks.

**Figure 3:**
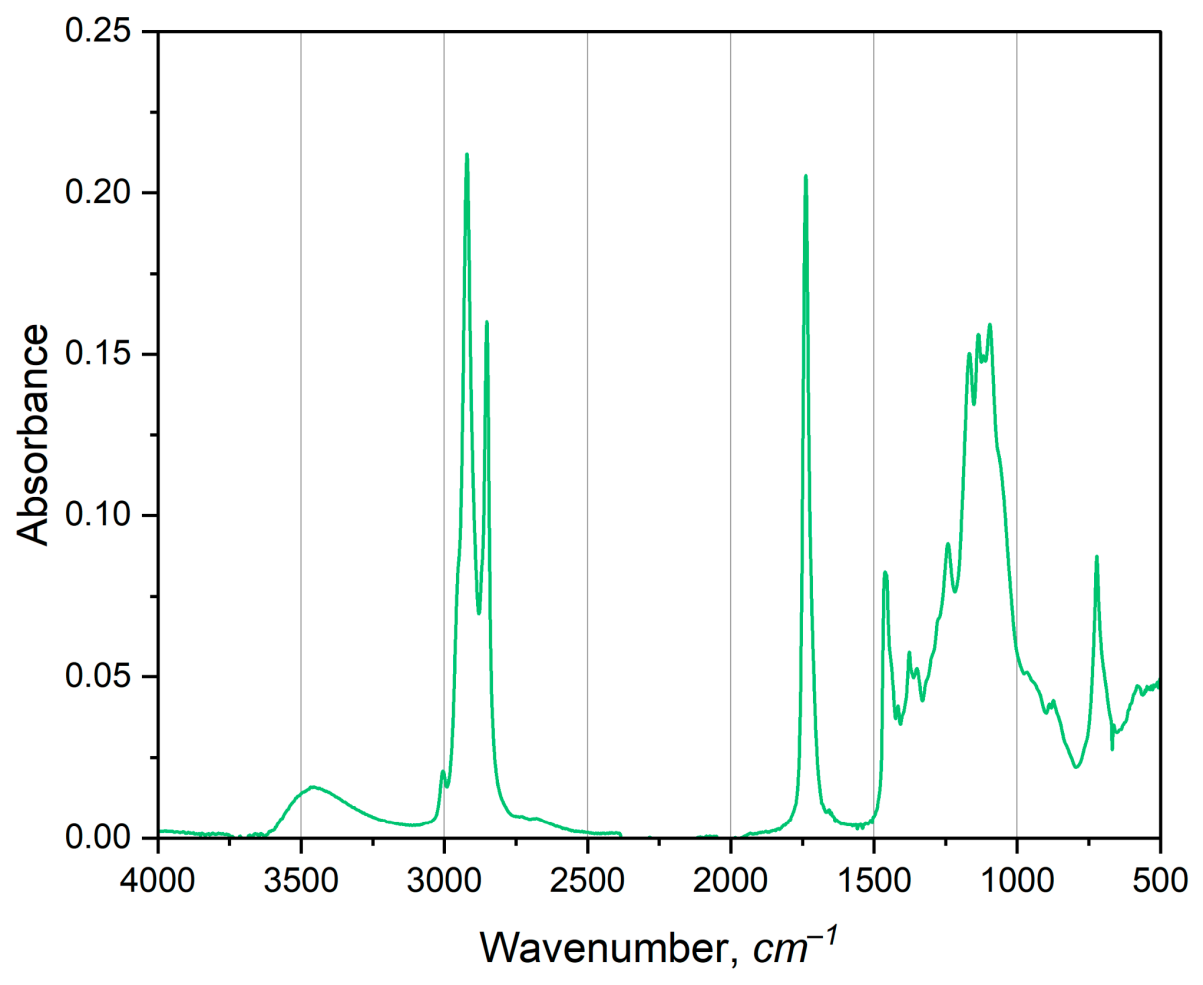
FTIR spectrum of biopolyol B.

Figure 3 shows the FTIR spectrum of biopolyol B. The broad band in the range 3600–3200 cm^−1^ is attributed to the stretching vibrations of the O-H groups coupled by hydrogen bonds. The substantial reduction in the characteristic C=C-related bands (3100 cm^−1^) indicates a high degree of conversion of the unsaturated groups during epoxidation. Narrow and intense bands in the wavenumber range 2920–2850 cm^−1^ correspond to the C-H stretching vibrations in the -CH2- and -CH3- groups associated with the aliphatic chains of fatty acid residues. A distinct band in the 1750–1730 cm^−1^ range originates from stretching vibrations of the carbonyl C=O groups in the esters, confirming the presence of ester bonds in the case of biopolyol B. The low intensity of the bands in the 1150–950 cm^−1^ range, typical of C-O-C vibrations in the oxirane ring, suggests a low content of epoxy groups. This observation was further confirmed by the determination of the epoxy value, which was 0.008 mol/100 g after the ring-opening reaction, indicating that only a small amount of unreacted epoxy groups remained in the product. The broad band in the 1150–1050 cm^−1^ range results from overlapping C-O stretching vibrations in ester and ether bonds, originating from both glycerol and DEG and structures formed after opening the oxirane rings. The absence of a distinct band in the 850–820 cm^−1^ range, attributed to C-H deformation vibrations in oxirane rings, indicates almost complete opening of oxirane rings by DEG. In the 900–700 cm^−1^ region, bands associated with C-H bending vibrations in aliphatic chains are observed, while signals below 700 cm^−1^ belong to the so-called fingerprint region, characteristic for a given substance.

### 3.2. Flexible Polyurethane Foam and Bio-Foam

Modification of FPUF with biopolyol B affected the foaming process of the polyurethane composition (Table 5). Composition F_B is characterized by longer start and gelation times than in the case of the reference system. Biopolyol B contains primary and secondary hydroxyl groups. The observed extension of the start and gelation times of the biopolyurethane composition after its application is related to the lower reactivity of secondary hydroxyl groups compared to primary ones. The growth time of the biocomposition is similar to that of REF. The curing time of F_B is 40% shorter than that of the reference composition. This phenomenon results from the increased proportion of hydroxyl groups introduced into the polyurethane composition along with the biopolyol B. A petrochemical polyol with an HV of 47 mgKOH/g is replaced with the biopolyol B characterized by a higher HV of 153 mgKOH/g. At a constant I_NCO_, this results in an acceleration of the polymer matrix crosslinking process. The polyurethane composition modified with the biopolyol B is characterized by lower maximum growth rate, maximum pressure, and a lower maximum core temperature of the growing foam than the reference system. The use of the biopolyol B with a high viscosity hinders the expansion of carbon dioxide bubbles in the polyurethane matrix, leading to a reduction in maximum pressure. The resulting biofoam has an approximately 13% higher shrinkage ratio than REF.

Table 6 presents sample SEM images and histograms of the cross-sectional area distribution of cells of the reference F_P and F_B foams. The distribution of pore cross-sectional areas presented in the histograms indicates that these foams are characterized by similar cellular morphology.

The results of the cell structure analysis presented in Table 7 confirm that the morphology of the F_P and F_B foams is very similar. The foams have the same cell count per mm^2^, cell anisotropy coefficient, as well as cell density. The cross-sectional area of the cells in the reference and biopolyol-modified foams is similar—the biofoam has the cell cross-sectional area of only about 4% larger than the unmodified foam.

FPUF modified with biopolyol B is characterized by a higher RS content (by 6%), apparent density (by 13%), hardness (by 36%), and hysteresis (by 10%) than the reference foam (Table 8). Biopolyol B is characterized by a higher hydroxyl group content compared to the petrochemical polyol it replaces. As its share in the reaction mixture increases, the total number of hydroxyl groups increases, which necessitates an increase in the amount of isocyanate used. Consequently, the RS share increases, while the share of long FS derived from the petrochemical polyol decreases. Increasing the RS share also leads to an increase in F_B hardness, and a decrease in the comfort factor and resilience of F_B.

### 3.3. Repolyols

The HV of the obtained FPUF repolyols ranges from 450 to 484 mgKOH/g, with a variation of approximately 7% between the recyclates (Table 9). This indicates that the obtained FPUF recyclates have similar HV values, regardless of the type of FPUF subjected to chemolysis. The AmV of FPUF repolyols modified with biopolyol is 3.9, which is slightly lower compared to the AmV of unmodified FPUF repolyol. The repolyol obtained from unmodified FPUF has a lower viscosity than the repolyol derived from FPUF containing the biopolyol B. Introducing the biopolyol B with a higher HV than the petrochemical polyol into the system during glycolysis promotes the formation of more branched and polar oligomers. The increased polarity and presence of ester bonds in rFB lead to intensified hydrogen bonding in the rebiopolyol, resulting in an increased viscosity compared to rFP. The densities of rFP and rEB_153_20 are similar, 1.10 and 1.09 g/cm^3^, respectively. The %DEG in rFP repolyol is 24.4%, while in rFB it reaches 23.8%. This means that the repolyol obtained from bio-based FPUF foam contains approximately 3% less DEG than the repolyol derived from unmodified foam. This phenomenon results from the higher content of ester bonds in the biopolyol used to obtain FPUF, which are more easily degraded by DEG. During glycolysis with DEG, this favours the formation of products with a higher proportion of oligomers and a slightly lower content of low-molecular-weight DEG.

Repolyol obtained from unmodified FPUF has higher average molecular weights, greater dispersity, and greater functionality compared to repolyol derived from bio-based FPUF foam (Table 8). Glycolysis of the reference FPUF produces longer oligomeric fragments than the recyclate bio-based FPUF. The bio-based FPUF used for synthesis has a lower average molecular weight and functionality, and introduces ester groups into the repolyol, which affects the DEG content of the rebio-based polyol.

**Figure 4:**
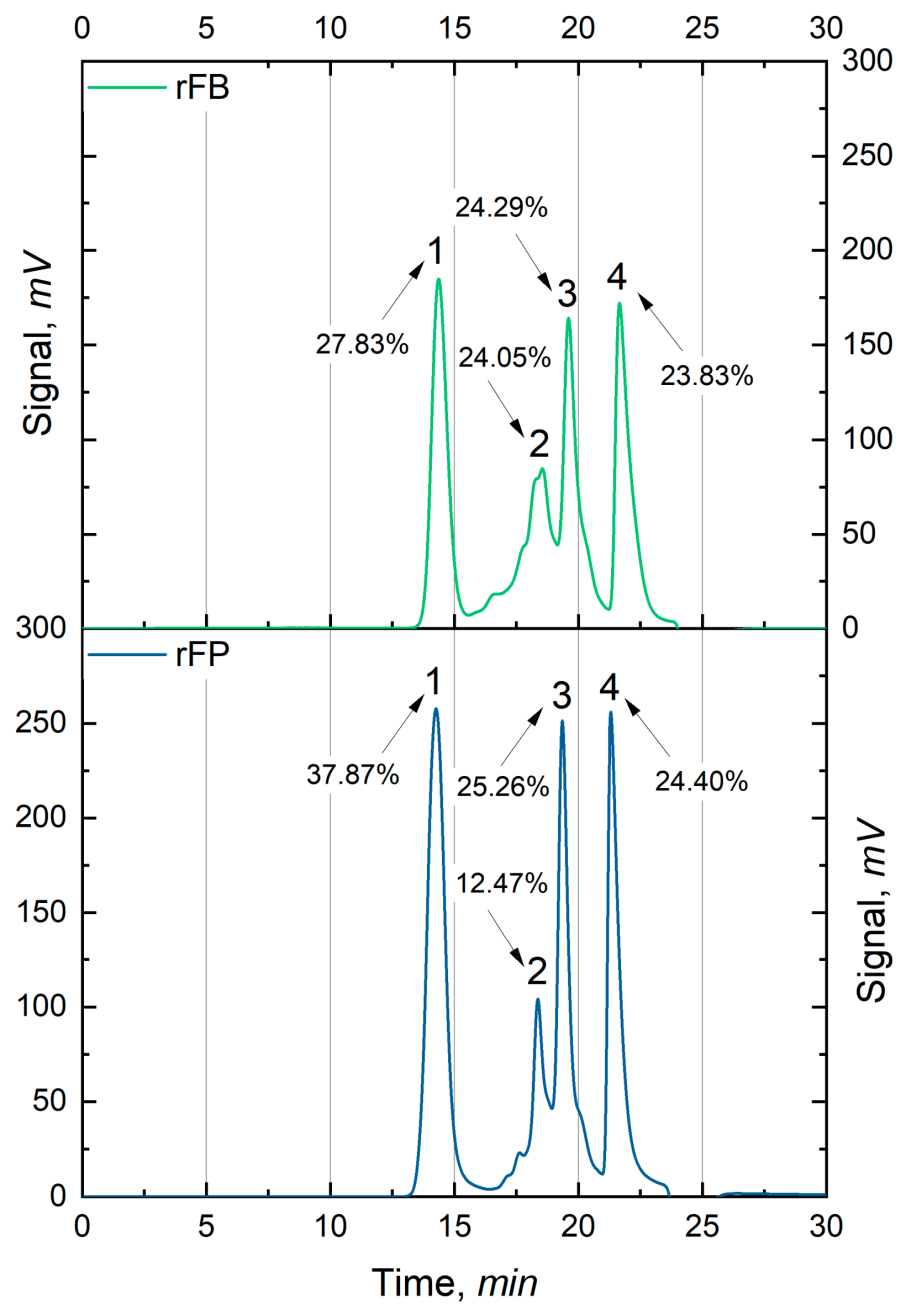
Chromatograms of FPUF repolyols.

GPC chromatograms indicate that FPUF repolyols exhibit a similar four-peak profile, suggesting a similar glycolysis mechanism. The use of biopolyol in recycled foam primarily affects the proportion of individual fractions without fundamentally changing the product’s character (Figure 4). Peak 1 corresponds to the oligomer fraction with the highest molecular weight. In the case of rFP, this peak is relatively narrow, while for the repolyol containing biopolyol, it broadens, indicating a broader molecular weight distribution resulting from the presence of fragments of both petrochemical and biopolyol origin. Peaks 2 and 3 represent oligomer fractions with medium and low molecular weights, resulting from partial degradation of the polyurethane network and reaction with DEG. Signal 4 is attributed to the presence of free, unreacted DEG used as the glycolysis agent. The graph shows the percentages of the surface areas of individual peaks.

**Figure 5:**
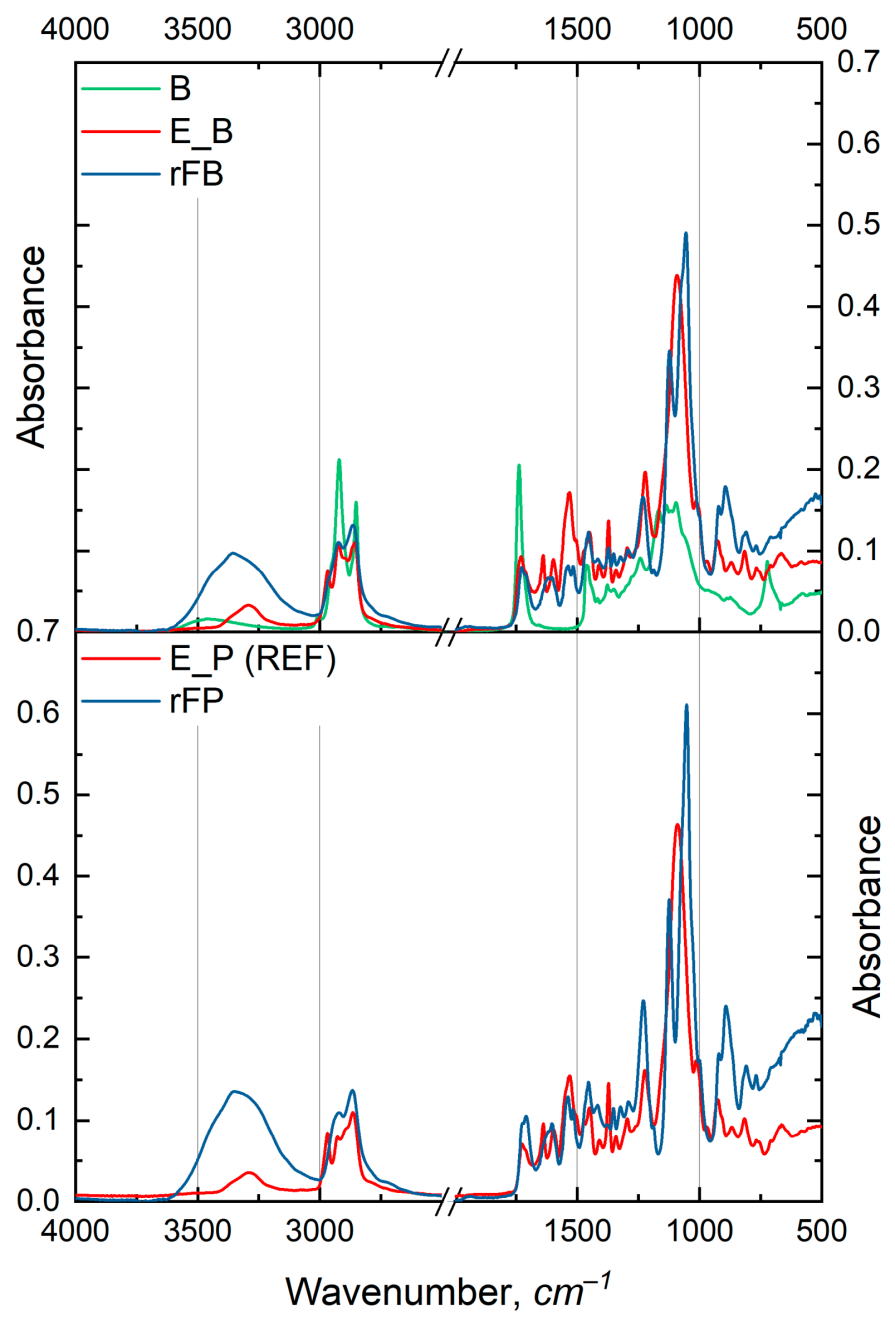
FTIR spectra of FPUF repolyols.

The FTIR spectra of both repolyols, shown in Figure 5, are highly similar, indicating similar chemical structures in both the reference repolyol and the biofoam-derived repolyol. The observed differences are primarily due to the presence of biopolyol fragments and the degree of urethane bond decomposition. The broad band in the 3600–3200 cm^−1^ range is attributed to O-H stretching vibrations associated with the presence of free hydroxyl groups, hydrogen-bonded hydroxyl groups, and free DEG. In the case of rFB, the signal is slightly broader, which can be attributed to the additional hydroxyl groups and ester groups that promote hydrogen bond formation. The bands in the 2950–2850 cm^−1^ range correspond to C-H stretching vibrations in the methyl and methylene groups of the aliphatic chains derived from the petrochemical polyol, biopolyol, and DEG. The signal in the 1730–1720 cm^−1^ range is associated with stretching vibrations of C=O bonds in ester groups, originating from both the biopolyol and the products formed during chemolysis. The band in the 1700–1660 cm^−1^ range is attributed to C=O vibrations of the urethane or urea residues, indicating incomplete cleavage of these bonds during chemolysis. In the 1500–1300 cm^−1^ region, bending C-H vibrations in aliphatic chains and weak signals associated with deformational N-H vibrations in urethane and urea groups are observed. Their low intensity results from the presence of polyurethane RS in the form of short fragments containing N-H and C=O groups. The intense bands in the 1150–1000 cm^−1^ range correspond to C-O-C stretching vibrations in the polyether structures of the polyol, biopolyol and DEG, and are similar in nature for both samples. This confirms the dominant contribution of polyether oligomers in the polyol composition. The 900–500 cm^−1^ range is a fingerprint region, encompassing C-C skeletal vibrations and C-H and C-O deformational vibrations characteristic of a given chemical.

### 3.4. Viscoelastic Polyurethane Foams

The obtained repolyols described in the previous section exhibit properties that allow us to propose their use as one of the polyol components for the production of polyurethane foams. Due to their high HV, these repolyols could not be used to produce classic flexible polyurethane foams. Therefore, recipes to produce viscoelastic foams were proposed, as polyols with higher HV can be used for their synthesis.

**Figure 6:**
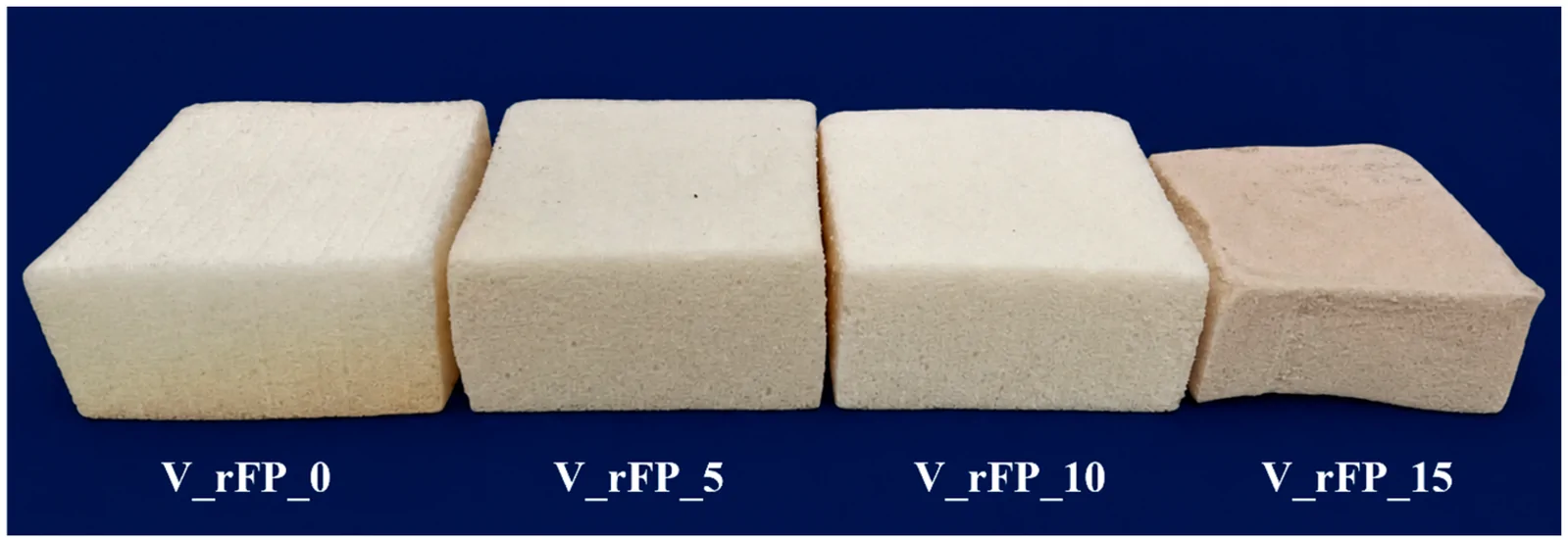
V_rFP photographs.

**Figure 7:**
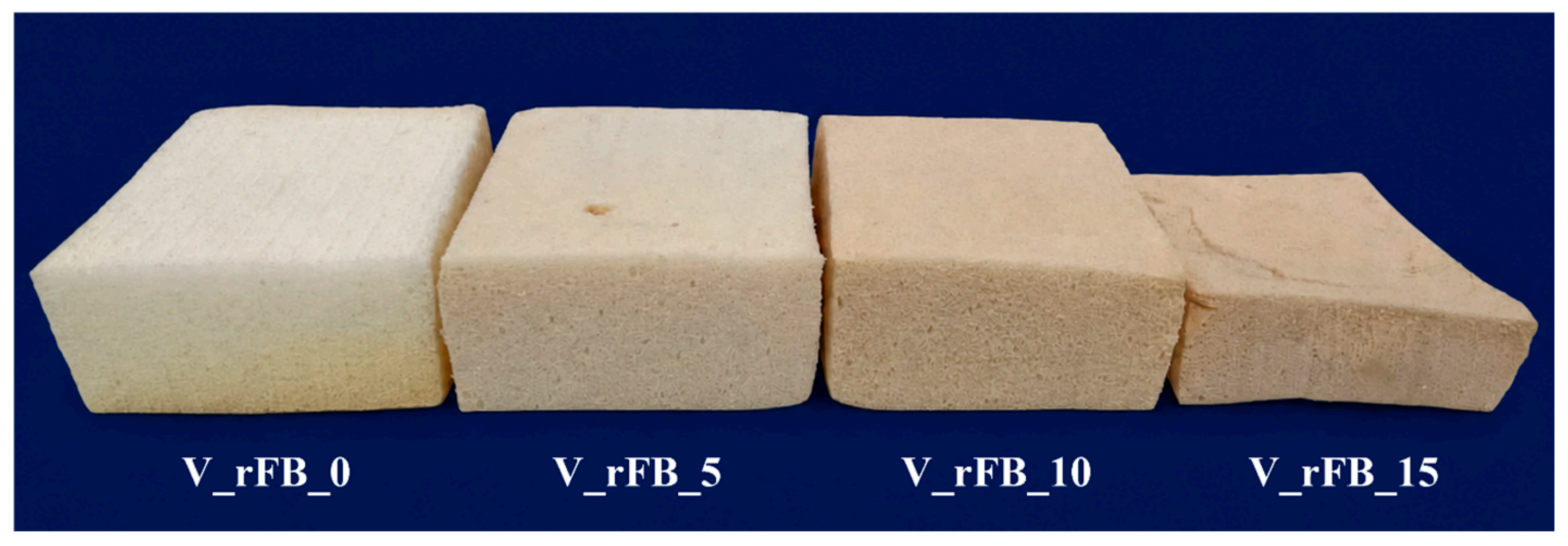
V_rFB photographs.

When 20% by weight of the aforementioned repolyols was used, the foams were observed to collapse shortly after rising, preventing further characterization of the material. Photographs of the resulting foams are included in Figure 6 and Figure 7.

**Figure 8:**
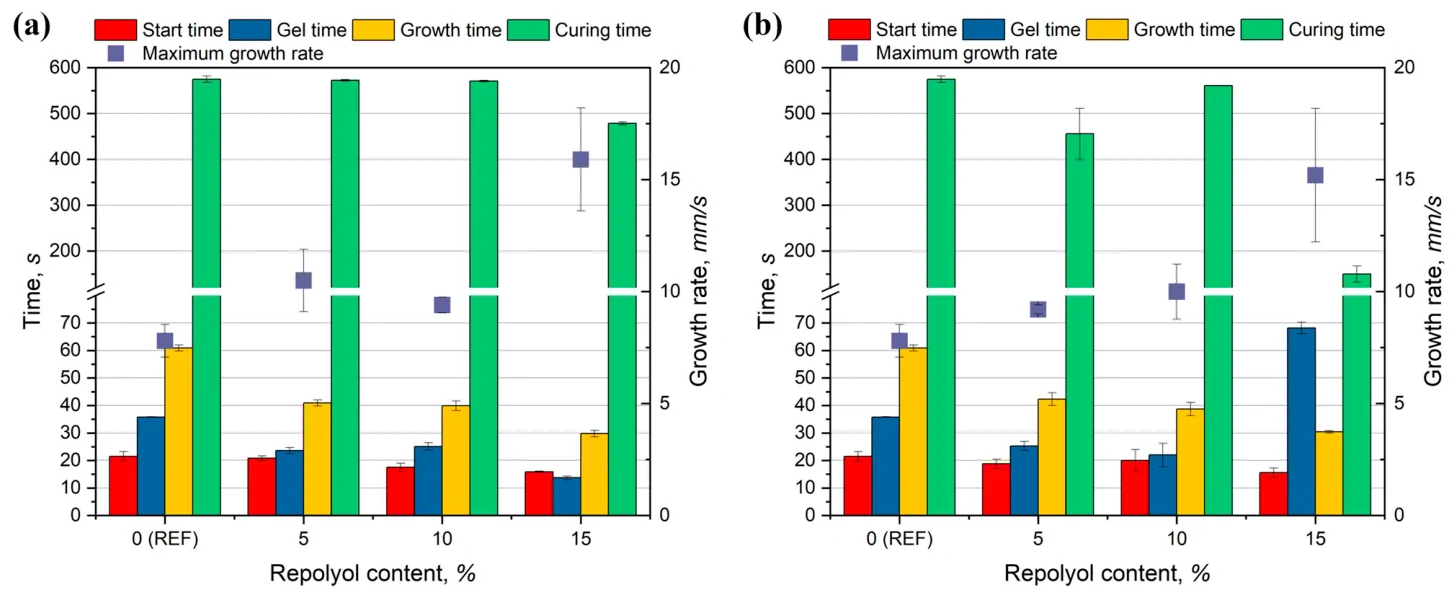
Characteristic times of the foaming process and the maximum growth rate of the compositions (**a**) V_rFP and (**b**) V_rFB.

The characteristic foaming times of systems modified with rFP and rFB repolyols show similar trends with their increasing mass fraction in the VPUF formulations (Figure 8). Increasing the contents of both types of repolyol in the polyurethane compositions leads to a slight shortening of the start time and a significant shortening of the rise time. This is due to the higher HV and AmV of the repolyols, which increases the system’s reactivity and accelerates the formation of urethane bonds. Furthermore, the lower viscosity of the repolyol compared to petrochemical polyols reduces the viscosity of the reaction mixture, facilitating reagent homogenization and blowing agent diffusion, which also shortens the start and rise times. Gradual increases in the rFP content in the polyurethane composition shorten the gelation time, which is a consequence of increasing the total HV and AmV, and thus increasing the system’s reactivity. In the case of the V_rFB series, a shortening of the gelation time is observed at 10 wt% repolyol addition, while for the V_rFB_15 composition, it is significantly extended. The use of repolyols in amounts up to 10 wt% does not significantly affect the curing time, especially considering the standard deviations. At 15 wt% rFP, its shortening is observed, while the use of 15 wt% rFB results in an almost four-fold reduction in the curing time. Higher repolyol contents increase the content of hydroxyl and amine groups, which, with a constant I_NCO_, promotes faster formation of a densely cross-linked polyurethane structure. This results in accelerated foam surface curing and a shortened curing time. In the case of rFB, this effect is stronger due to the higher HV and the presence of low-molecular-weight fragments (DEG residues), which increase the mobility of the chains and intensify the cross-linking process, leading to a faster increase in the system viscosity and earlier curing of the foam surface. The maximum foam growth rate increases with increasing the repolyol content. The greatest differences from the reference material are observed for samples containing 15% by weight of repolyols, whose growth rate increases by approximately 50%. This phenomenon results from the introduction of additional hydroxyl and amine groups, which intensify the chemical interactions, as well as the presence of DEG, which are more mobile than longer polyol chains. Maintaining a constant I_NCO_ leads to an increased rate of gas generation and matrix gelation, with this effect being most pronounced for the 15% by weight modification of the polyol system.

The maximum core temperature of all VPUF foams modified with repolyols exceeds that of the reference material and increases with increasing recyclate content in the formulations (Table 10). The highest values of this parameter were obtained for V_rFP_15 and V_rFB_15 foams. The use of repolyols results in the introduction of a greater value of hydroxyl groups into the system compared to the petrochemical polyols they replace. Furthermore, the presence of amine groups and low-molecular-weight fragments derived from DEG increases the reactivity of the reaction mixture. This results in an intensification of the exothermic process of urethane bond formation, which is manifested by an increase in the core temperature during foam growth. At 15% by weight of repolyol, the system achieves the highest reactivity, leading to the rapid release of large amounts of heat and, consequently, the highest maximum temperature. A similar trend is observed for the maximum pressure inside the cells of the growing foams—all modified materials are characterized by higher values of this parameter than the reference foam. At the same time, foams containing 10% by weight of repolyols in the polyol component are characterized by the lowest maximum pressure among the systems modified with chemolysis products. The introduction of components with a larger value of hydroxyl and amine groups, in addition to DEG, promotes an increased rate of blowing gas generation. In the case of the V_rFP_10 and V_rFB_10 systems, the chemical reactions and the increase in viscosity are relatively balanced, allowing for more free gas expansion and resulting in a slight reduction in the maximum pressure in the foam pores. Changes in the VPUF shrinkage ratio correlate with the pressure and maximum temperature of the foam core. All materials modified with repolyols are characterized by greater shrinkage than the reference system, and its value increases with the increase in the share of recyclates in the formulation. The presence of repolyols in PU compositions can increase their viscosity and increase the crosslinking density of the final foam material. This hinders cell opening during VPPU growth and crosslinking. Pressurized foaming gas remains in the partially closed foam pores. As the exothermic foaming and gelation reaction ends, the foam temperature decreases. This reduction in temperature reduces the pressure within the closed cells. Consequently, this leads to increased foam shrinkage with increasing repolyol content.

**Figure 9:**
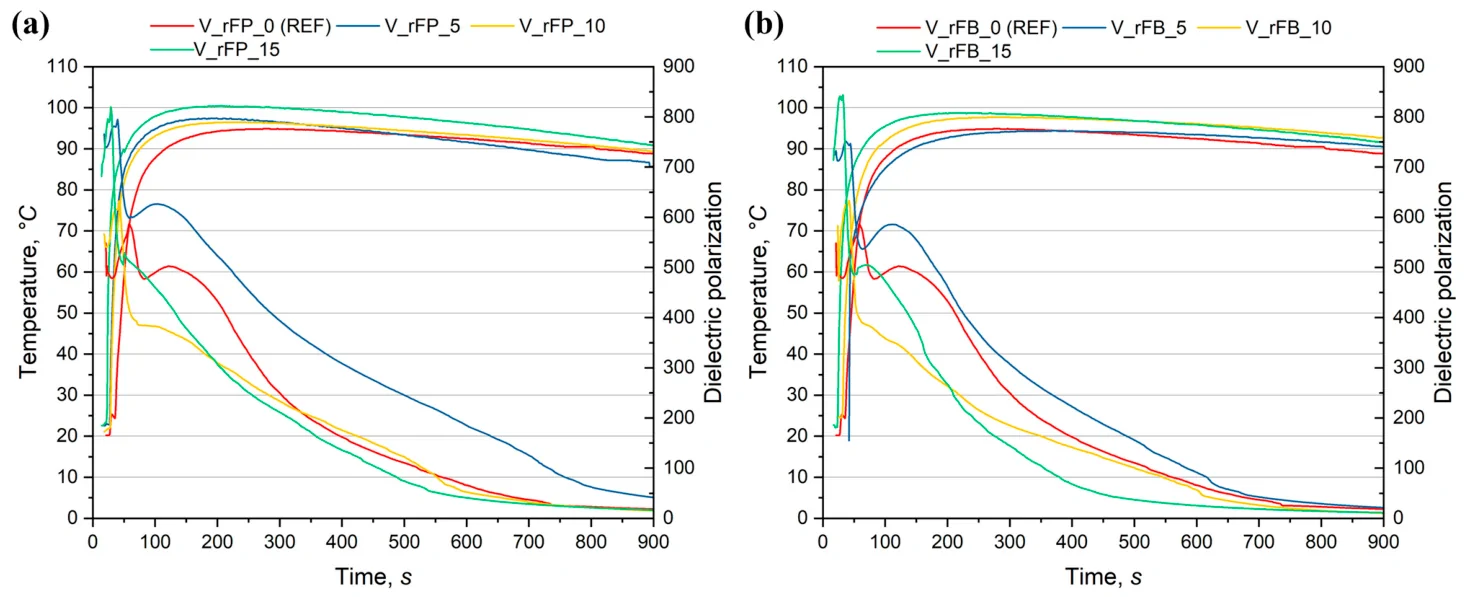
Curves of temperature and dielectric polarization changes during the foaming process of the compositions (**a**) V_rFP and (**b**) V_rFB.

The core temperature curves of the growing VPUF foams follow a very similar pattern, regardless of the type and amount of FPUF repolyol used (Figure 9). In the systems modified with rFP and rFB repolyols, the lowest temperature values after 900 s are observed for the V_rFP_5 and V_rFB_5 compositions, respectively. Analysis of the dielectric polarization changes over time indicates its transient increase in the 100–150 s range for both the reference system and the compositions containing the lowest amounts of repolyols. This phenomenon is related to the presence of uncrosslinked or weakly crosslinked polymer chain fragments in the liquid phase, which have not yet been incorporated into the polyurethane network structure. This effect is less pronounced in compositions containing 10% by weight and 15% by weight of repolyol in the polyol component, which results from a faster increase in the viscosity and degree of crosslinking of the reaction system. Consequently, in the 100–150 s range, the proportion of free, polarized molecules decreases, and the observed polarization maximum is lower. Systems in which 15 wt% of the petrochemical polyols were replaced with rFP and rFB repolyols are characterized by the fastest reduction in dielectric polarization, indicating their highest reactivity. An exception is the V_rFP_5 composition, for which the dielectric polarization does not reach a value close to zero after 900 s of polarization. A small addition of rFP introduces highly polar segments into the system, such as amino groups and low-molecular-weight DEG, which partially remain outside the network or are very weakly bound to it, retaining the ability to orient in the electric field even after the gelation phase.

Among the VPUF foams modified with rFP repolyol, V_rFP_10 has the most uniform cell morphology, while V_rFP_15 is characterized by the largest number of pores with the smallest cross-sectional area (Table 11). The structural uniformity of V_rFP_10 results from the favourable 10 wt% repolyol content in the polyol system, which provides a compromise between increased reactivity (related to the higher content of hydroxyl and amine groups in the PU formulation) and rheological properties and gelation time. These conditions favour stable and uniform foam cell development. In contrast, the V_rFP_15 system exhibits greater reactivity and viscosity, leading to accelerated gas generation and faster gelation of the polyurethane network.

Similar to the V_rFP series foams, the V_rFB_10 composition has the highest degree of cellular structure homogeneity (Table 12). In turn, the V_rFB_15 foam is characterized by the most developed fine porosity, which is consistent with the observations for the V_rFP systems. The mechanisms responsible for these relationships are identical and were discussed in the previous paragraph.

As the mass fraction of rFP repolyol in VPUF increases, the number of cells per 1 mm^2^ and the cell density of the foams increases (Table 13). Simultaneously, the cross-sectional area of the cells decreases, while the anisotropy coefficient remains unchanged. The repolyol with higher reactivity, containing a higher proportion of short DEG, promotes intensification of the nucleation process and faster stabilization of small cells, without significantly affecting their growth direction. Composition V_rFP_15 has cell structure parameters most similar to the reference foam, resulting from achieving a balance between the increased reactivity of the system (manifested by a higher core temperature and higher pressure in the cells), the viscosity of the reaction mixture, and the gelation rate. As a result, the accelerated nucleation is compensated by faster formation of the polyurethane network, leading to a structure most similar to the reference system. Increasing the mass fraction of rFB repolyol in the VPUF does not cause significant changes in the number of cells per 1 mm^2^ or their cross-sectional area (Table 13). However, these values remain approximately 45% and 50% higher, respectively, compared to the reference system. V_rFB_10 has a cell anisotropy coefficient identical to the reference foam. Furthermore, the cell density of all foams modified with rFB repolyol does not exceed 60% of the value characteristic for the reference system.

Gradual replacement of petrochemical polyols with increasing mass fractions of rFP and rFB repolyols leads to a reduction in the FS content in the foam morphology and an extension of their recovery time (Table 14). In the case of V_rFP_15 and V_rFB_15 compositions, the recovery time exceeds 300 s. Increasing the repolyol content results in a reduction in the proportion of long, petrochemically derived FS fragments in favor of shorter, stiffer, and polar molecules, such as DEG or amine groups. An increased RS content and enhanced physical interactions, particularly hydrogen bonds, limit the rate of strain relaxation after unloading, which manifests itself in a greater susceptibility of the material to permanent deformation.

**Figure 10:**
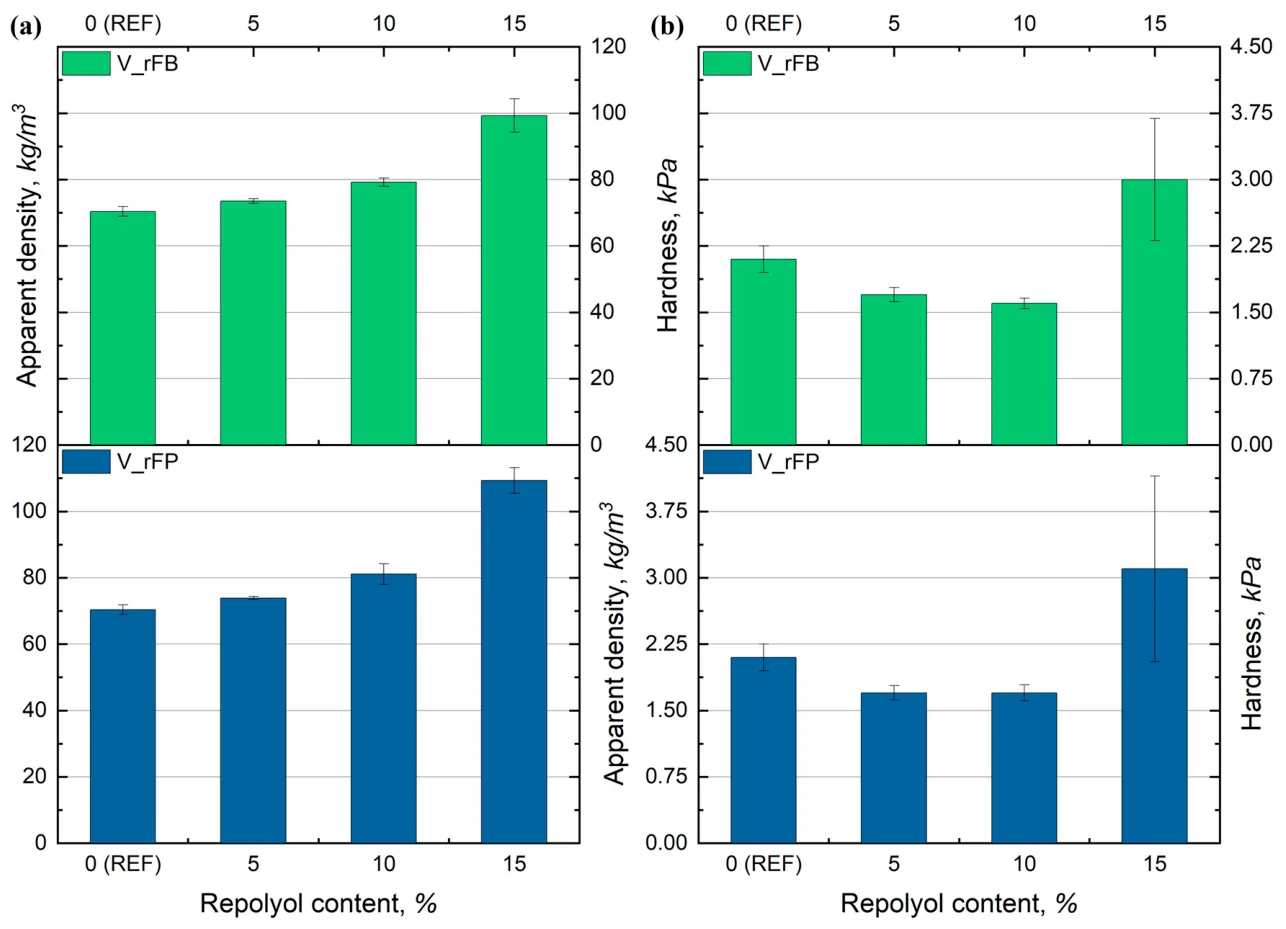
Apparent density (**a**) and hardness (**b**) of V_rFP and V_rFB.

The effects of increasing the mass fraction of rFP and rFB repolyols in VPUF formulations on the apparent density of the foams are similar (Figure 10). Increasing their content systematically increases the apparent density. This phenomenon results from the greater exotherm of the synthesis process and the acceleration of the foaming and gelation stages of the polyurethane system, which limits the possibility of free gas expansion in the pore structure. Higher reactive repolyols, containing a larger proportion of short chains and polar segments, increase the mixture viscosity and shorten the time to form the polymer network. Consequently, gas expansion is hindered, which promotes the formation of a fine-cell structure and translates into an increase in the apparent density of the foams. The highest values of this parameter were recorded for the V_rFP_15 and V_rFB_15 compositions, which are 35% and 29% higher, respectively, compared to the reference system.

Partially replacing petrochemical polyols with FPUF-derived repolyols up to 10 wt% in VPUF formulations resulted in a reduction in foam hardness (Figure 10). Increasing the repolyol content to 15 wt% resulted in a significant increase in hardness, exceeding that of the reference foam by approximately 31%. The increase in apparent density observed for the foams containing 15 wt% repolyol should also be considered when interpreting their mechanical properties, as higher density may contribute to increased hardness. However, the relationship between apparent density and hardness was not linear across the investigated formulations. For example, for V_rEP, the apparent density increased from 70.4 to 81.1 kg/m^3^ between 0 and 10 wt% repolyol, whereas hardness decreased from 2.1 to 1.7 kPa. A similar trend was observed for V_rEB, where the density increased from 70.4 to 79.2 kg/m^3^, while hardness decreased from 2.1 to 1.6 kPa. Therefore, although the higher density of the 15 wt% formulations may contribute to their increased hardness, the observed increase cannot be attributed solely to density. At the same time, the presence of a higher proportion of short, relatively stiff and polar segments may promote stronger intermolecular interactions, including hydrogen bonding, which may additionally contribute to the observed mechanical response. Thus, the increased hardness of the 15 wt% formulations is likely to result from a combined effect of foam density and changes in the polymer structure and intermolecular interactions. However, the present results do not allow these individual contributions to be quantitatively separated.

**Figure 11:**
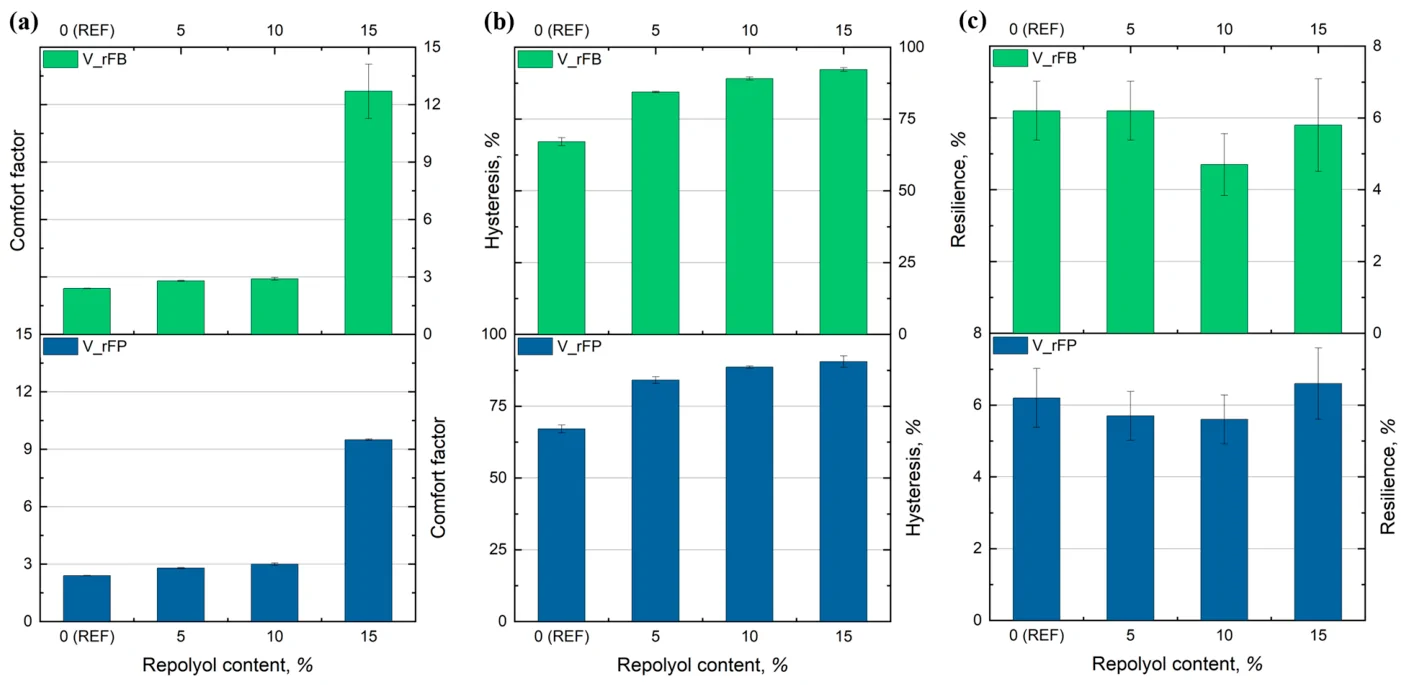
Comfort factor (**a**), hysteresis (**b**) and resilience (**c**) of V_rFP and V_rFB.

Increasing the mass fraction of rFP and rFB repolyols in VPUF formulations resulted in similar changes in the comfort factor of the foams (Figure 11). Replacing petrochemical polyols with these repolyols up to 10 wt% resulted in only a slight increase in the comfort factor. The moderate increase in the content of short, more polar segments may affect intermolecular interactions, including hydrogen bonding, while maintaining a relatively high degree of material elasticity. However, further increasing the repolyol fraction to 15 wt% resulted in a pronounced increase in the comfort factor. For the V_rFP_15 and V_rFB_15 formulations, the comfort factor values were approximately four- and five-fold higher, respectively, than that of the reference system. Importantly, the increase in comfort factor at 15 wt% repolyol was substantially greater than the corresponding increase in apparent density, suggesting that the observed change in viscoelastic behavior cannot be fully explained by density alone.

The pronounced increase in the comfort factor observed at 15 wt% repolyol may be related to changes in the polymer structure and intermolecular interactions associated with the increased proportion of short, polar segments. The simultaneous changes in apparent density, rigid-segment fraction (RS), and recovery time may also contribute to the observed mechanical response. These factors may affect the resistance of the foam to deformation, particularly at higher deformation levels, and consequently contribute to the increased comfort factor. However, since the crosslink density was not directly determined in the present study, the observed changes cannot be conclusively attributed to an increased degree of crosslinking. The possible contribution of changes in the polymer network and intermolecular interactions is therefore considered a proposed mechanism rather than a directly demonstrated effect.

Similarly to the comfort factor, increasing the mass fraction of rFP and rFB repolyols in VPUF formulations leads to similar changes in the hysteresis values of the foams (Figure 11). The most pronounced increase occurs when moving from the reference system to foams in which 5% by weight of petrochemical polyols are replaced by repolyols, with differences reaching approximately 20%. A small addition of repolyols introduces short, highly polarized chain fragments into the foam structure, originating, among other things, from the residues of unreacted DEG present in the recyclates, resulting in increased energy losses during loading and unloading cycles. Further increases in the repolyol content no longer result in such a significant increase in hysteresis. Although the polyurethane matrix becomes more crosslinked and stiffer, the mobility of both the FS and the stiff polyols is limited, which favours a more elastic response of the material to deformation and, consequently, does not lead to a further significant increase in hysteresis.

The resilience of VPUF foams modified with rFP repolyol decreases with increasing mass fraction (Figure 11). This phenomenon is a consequence of increased energy losses during load and unload cycles, meaning that less of it is recovered as rebound. This is due to the nature of the foam morphology, which increases the proportion of short, polar chain segments and the intensity of intermolecular interactions. A deviation from this trend is the V_rFP_15 foam, for which the resilience is approximately 6% higher than in the reference system. In this case, the higher apparent density favours partial energy recovery, resulting in a slight increase in resilience. At the same time, the presence of short, polar fragments in the polyurethane structure and increased hysteresis result in a greater ability of the material to absorb energy, which translates into lower resilience of the V_rFB foams compared to the reference system.

**Figure 12:**
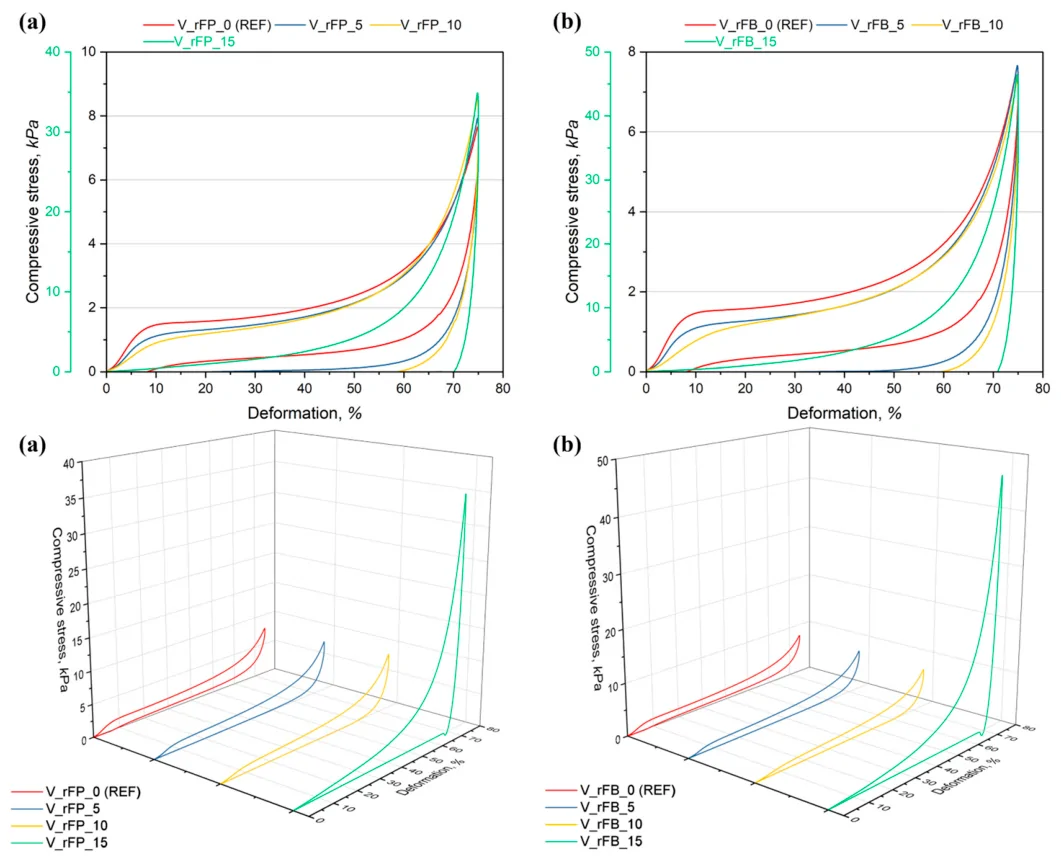
Hysteresis loops for (**a**) V_rFP and (**b**) V_rFB.

The hysteresis loop curve shown in Figure 12 indicates that partial replacement of petrochemical polyols with rFP repolyol at 5% and 10% by weight leads to a slight reduction in the stiffness of the foams at low and medium strains and to an increase in the loop area, which corresponds to greater energy losses during loading and unloading cycles. This effect may result from the introduction of shorter, more polar chain fragments, which intensify intermolecular interactions, increasing internal friction and material hysteresis. However, for the V_rFP_15 composition, a significantly steeper loading curve slope and significantly higher compressive stresses at 75% strain are observed. This indicates an increase in the stiffness of the system, associated with higher apparent density of the foams, which limits the mobility of polymer segments.

As the rFB repolyol content in the polyol component increases, a shift in the load-unload curves toward lower stress values at a given deformation is observed (Figure 12). At the same time, the hysteresis loops become wider, indicating greater energy dissipation during the loading–unloading cycle. A larger hysteresis loop area corresponds to increased energy losses and, consequently, lower energy recovery from the deformation cycle. The introduction of rFB into the VPUF formulation results in an increased proportion of short, polar chain segments. Consequently, the material exhibits greater energy dissipation during deformation, manifested by the increased hysteresis loop area.

## 4. Conclusions

Studies have shown that new-generation VPUFs can be effectively produced with appropriately selected repolyols derived from chemically recycled FPUFs and bio-based foams, in line with the principles of the Circular Economy. For the first time, the feasibility of using repolyol obtained from the glycolysis of flexible polyurethane foam containing biopolyol for the synthesis of new-generation viscoelastic foams has been demonstrated.

The introduction of repolyols in various mass fractions results in VPUFs with up to 24% lower hardness and increased apparent density, while maintaining comparable cellular morphology and characteristic slow recovery after the deformation force is removed. SEM analysis shows that the use of repolyols affects the cell size distribution without reducing the mechanical strength of the foams.

The physicomechanical properties of the foams demonstrate that appropriately designed formulations containing repolyols enable the production of VPUFs that retain the functional properties required for conventional VPUFs derived from petrochemical polyols. Foams containing repolyols exhibit up to 27% greater energy absorption capacity than unmodified materials. The tested solutions enable the production of new-generation VPUFs, where chemical recycling is not merely a waste management method but also enables the production of high-quality raw materials used in chemical syntheses.

From an application and environmental perspective, the resulting foams demonstrate properties that suggest potential for use in the furniture and mattress industries, while simultaneously increasing the share of recycled raw materials. Effective use of repolyols in VPUF formulations allows for reduced fossil fuel consumption and the production of sustainable and circular polyurethanes. Furthermore, the obtained results demonstrate the need for further refinement of repolyol and foam production conditions to ensure consistent raw material properties and maximize polyurethane waste recovery.

## Figures and Tables

**Table 1:** FPUF formulations.

		F_P * (REF)	F_B
	
Petrochemical polyol Rokopol^®^ F3600, g		100	80
Biopolyol B, g		0	20
Blowing agent H_2_O, g		3.02	2.98
Catalyst Dabco^®^ T-9, g		0.10	0.10
Catalyst Dabco^®^ BLV, g		0.14	0.14
Surfactant Vorasurf^TM^ DC 5906, g		1.00	1.00
I_NCO_		0.89	0.89

Note: F—flexible foam; B—biopolyol with LOH 153 mgKOH/g; *—amount of biopolyol replacing petrochemical polyol in the polyol component.

**Table 2:** V_rFP formulations.

		V_rFP_0 * (REF)	V_rFP_5	V_rFP_10	V_rFP_15	V_rFP_20
	
Petrochemical polyol Rokopol^®^ RF551, g		40.0	37.5	35.0	32.5	30.0
Petrochemical polyol Rokopol^®^ M6000, g		40.0	37.5	35.0	32.5	30.0
Petrochemical polyol Rokopol^®^ M1170, g		20.0	20.0			
Repolyol rFP, g		0	5	10	15	20
Blowing agent H_2_O, g		2.50	2.50			
Catalyst Dabco^®^ DC 2, g		1.00	1.00			
Surfactant Niax^TM^ Silicone L-627, g		0.80	0.80			
I_NCO_		0.45	0.45			

Note: V—viscoelastic foam; rFP—EPPU repolyol with LOH 450 mgKOH/g; *—amount of repolyol replacing petrochemical polyol in the polyol component.

**Table 3:** V_rFB formulations.

		V_rFB_0 * (REF)	V_rFB_5	V_rFB_10	V_rFB_15	V_rFB_20
	
Petrochemical polyol Rokopol^®^ RF551, g		40.0	37.5	35.0	32.5	30.0
Petrochemical polyol Rokopol^®^ M6000, g		40.0	37.5	35.0	32.5	30.0
Petrochemical polyol Rokopol^®^ M1170, g		20.0	20.0			
Repolyol rFB, g		0	5	10	15	20
Blowing agent H_2_O, g		2.50	2.50			
Catalyst Dabco^®^ DC 2, g		1.00	1.00			
Surfactant Niax^TM^ Silicone L-627, g		0.80	0.80			
I_NCO_		0.45	0.45			

Note: V—viscoelastic foam; rFB—EPPU repolyol with LOH 484 mgKOH/g; *—amount of repolyol replacing petrochemical polyol in the polyol component.

**Table 4:** Physicochemical properties of biopolyol.

Property	Value
Hydroxyl value, mgKOH/g	153
Acid value, mgKOH/g	9.9
Epoxide value, mol/100 g	0.008
Viscosity, mPa·s	440
Density, g/cm^3^	0.97
Water content (%H_2_O), %	0.29
Number average molecular weight (Mn), g/mol	1057
Weight average molecular weight (Mw), g/mol	1251
Molecular weight dispersity (d)	1.2
Functionality (f)	2.9

**Table 5:** Results of the analysis of the foaming process of FPUF polyurethane compositions.

Property	Value	
	F_P (REF)	F_B
Start time, s	24 ± 5.2	30 ± 0.5
Gel time, s	21 ± 2.2	27 ± 0.3
Rise time, s	333 ± 22.1	310 ± 3.5
Curing time, s	736 ± 22.1	439 ± 5.7
Maximum growth speed, mm/s	2.7 ± 0.51	0.8 ± 0.04
Maximum temperature, °C	96 ± 6.1	74 ± 1.1
Maximum pressure, Pa	65 ± 6.4	16 ± 2.1
Shrinkage ratio, %	2.8 ± 0.14	3.2 ± 0.0

**Table 6:** SEM images and histograms of FPUF cell surface areas.

Foam Symbol	SEM Photo	Histogram
F_P (REF)	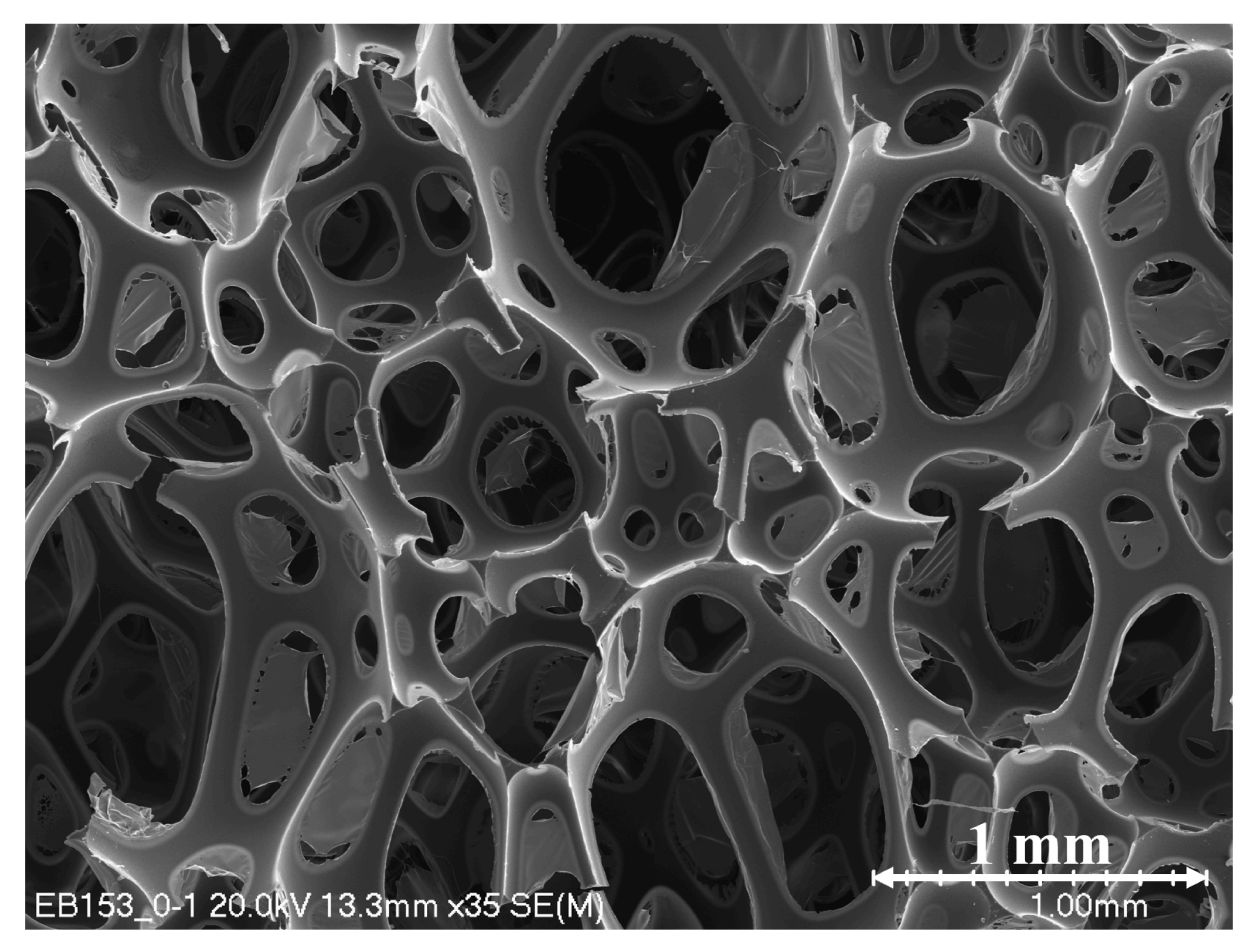	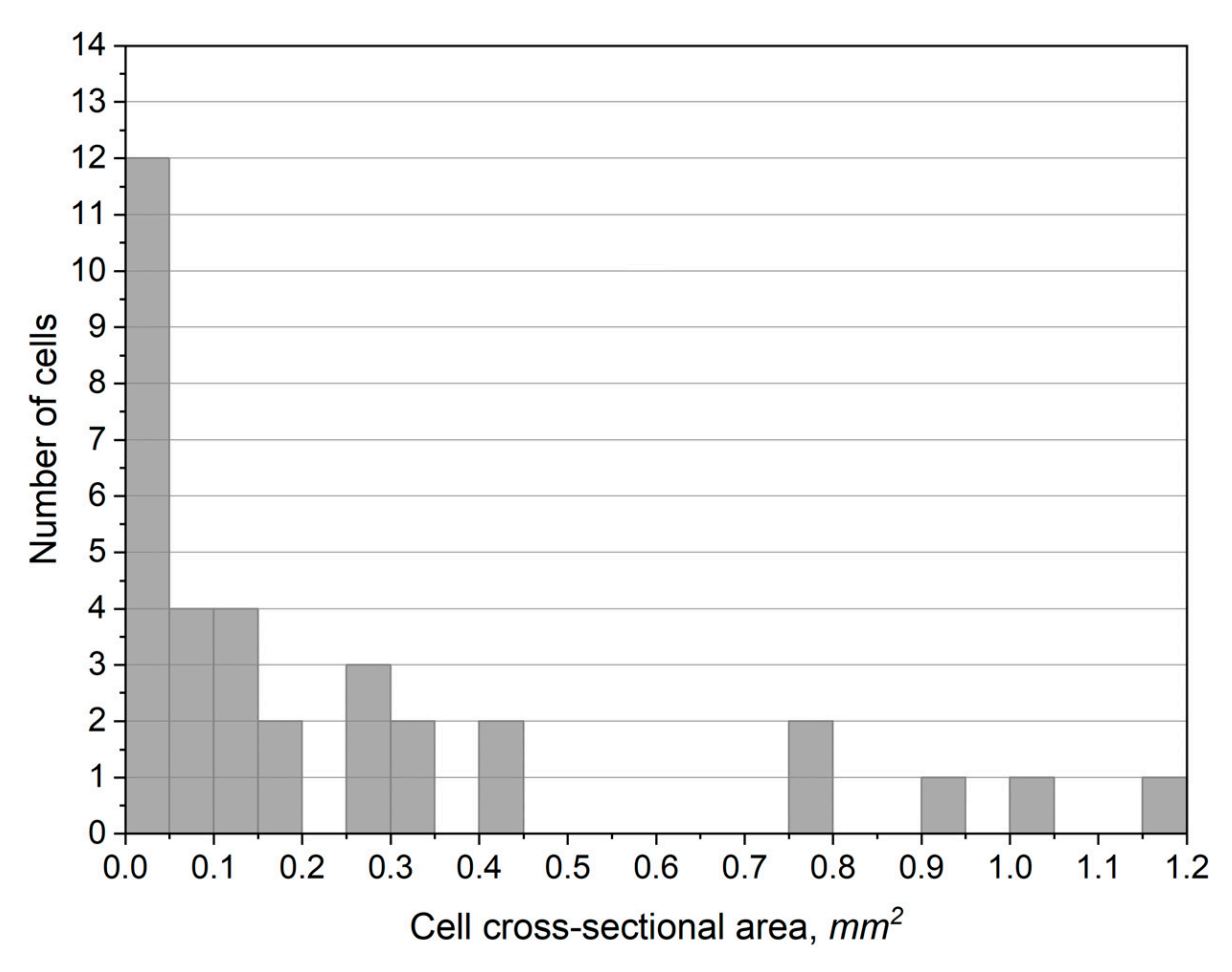
F_B	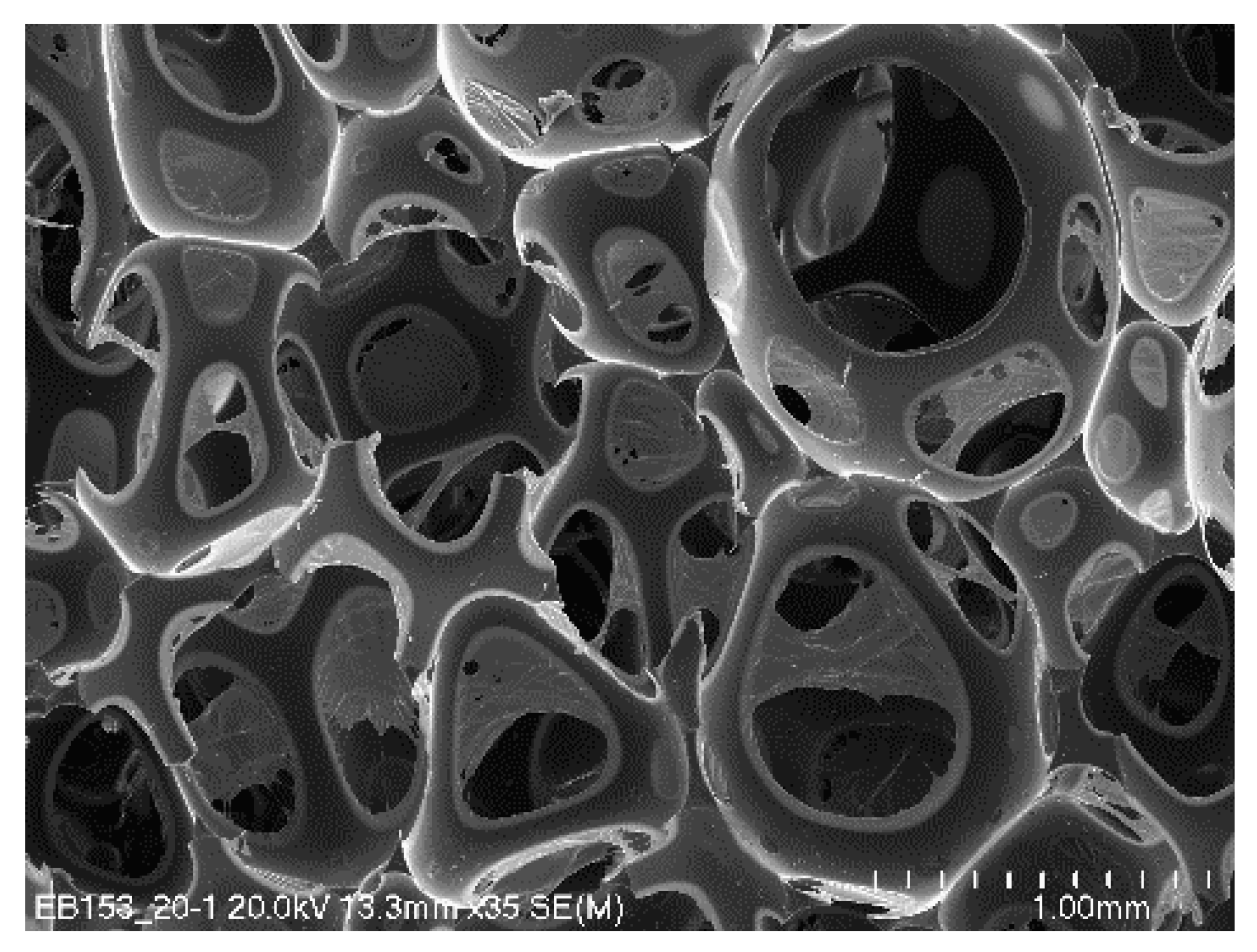	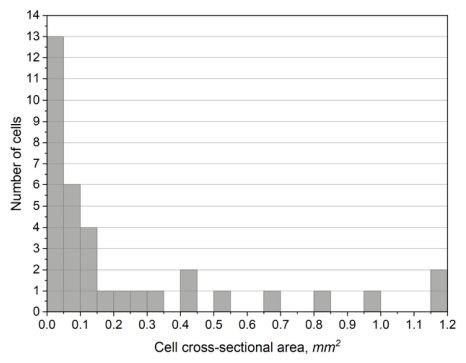

**Table 7:** Results of the analysis of the FPUF cellular structure.

Property	Value	
	F_P (REF)	F_B
Number of cells per 1 mm^2^	4 ± 0.4	4 ± 0.3
Cell cross-sectional area, mm^2^	0.22 ± 0.021	0.23 ± 0.018
Cell anisotropy coefficient	1.1 ± 0.05	1.1 ± 0.13
Cell density, cells/mm^3^	7 ± 1.0	7 ± 0.8

**Table 8:** Physicomechanical properties of FPUF.

Property	Value	
	F_P (REF)	F_B
Flexible segments, %	75.3	73.7
Rigid segments, %	24.7	26.3
Apparent density, kg/m^3^	43.8 ± 0.84	50.3 ± 0.09
Hardness, kPa	5.0 ± 0.05	7.8 ± 0.20
Hysteresis, %	40.4 ± 0.52	44.9 ± 0.74
Comfort factor	2.4 ± 0.02	2.2 ± 0.01
Resilience, %	39.8 ± 2.61	36.7 ± 0.99

**Table 9:** Physicochemical properties of FPUF repolyols.

Property	Value	
	rFP	rFB
Hydroxyl value, mgKOH/g	450	484
Amine value, mgKOH/g	4.6	3.9
Viscosity, mPa·s	776	910
Density, g/cm^3^	1.10	1.09
DEG content (%DEG), %	24.4	23.8
Number average molecular weight (Mn), g/mol	317	277
Weight average molecular weight (Mw), g/mol	864	706
Molecular weight dispersity (d)	2.7	2.6
Functionality (f)	2.5	2.4

**Table 10:** Maximum temperature, maximum pressure and shrinkage ratio of the V_rFP and V_rFB compositions.

Foam Symbol	Maximum Temperature, °C	Maximum Pressure, Pa	Shrinkage Ratio, %
V_rFP_0/V_rFB_0 (REF)	93 ± 3.9	213 ± 21.2	1.6 ± 0.06
V_rFP_5	97 ± 0.3	533 ± 26.2	2.5 ± 0.35
V_rFP_10	97 ± 0.2	450 ± 7.5	3.6 ± 0.32
V_rFP_15	100 ± 0.9	508 ± 26.9	5.8 ± 0.00
V_rFB_5	95 ± 1.2	597 ± 9.2	2.7 ± 0.00
V_rFB_10	98 ± 0.7	472 ± 39.5	4.1 ± 0.91
V_rFB_15	108 ± 12.9	579 ± 29.7	7.4 ± 0.49

**Table 11:** SEM images and histograms of V_rFP cell areas.

Foam Symbol	SEM Photo	Histogram
V_rFP_0 (REF)	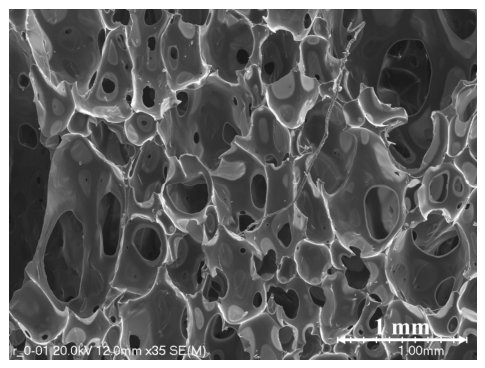	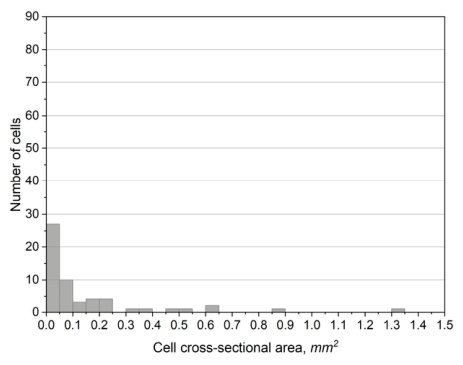
V_rFP_5	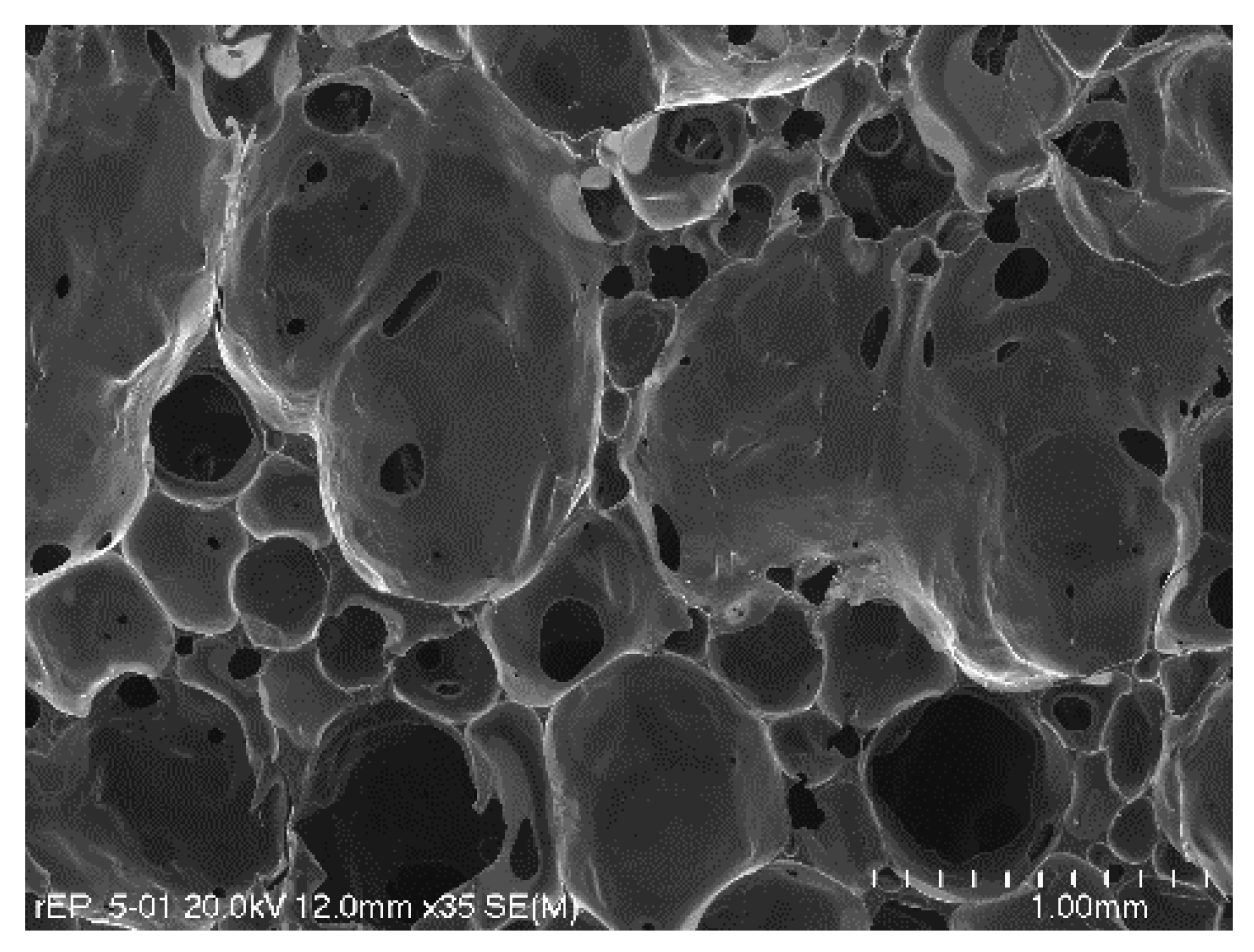	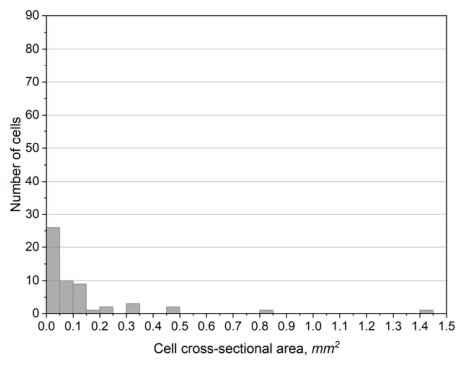
V_rFP_10	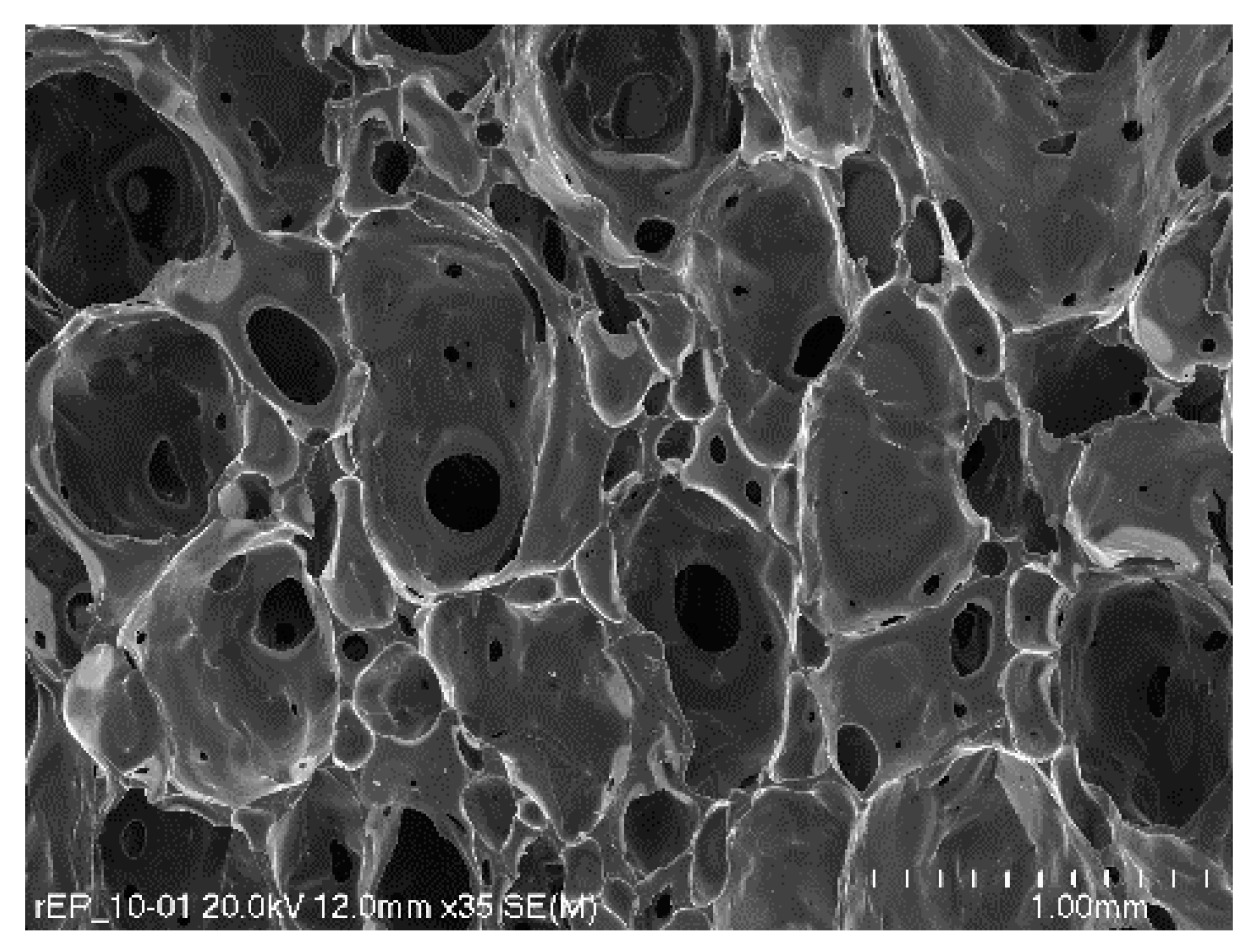	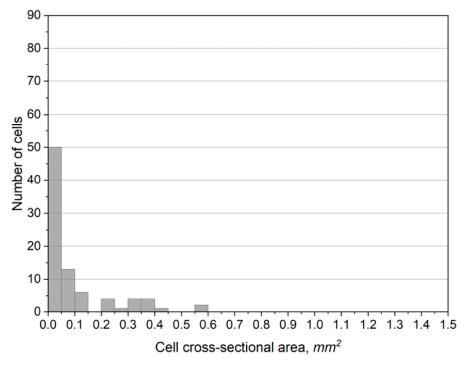
V_rFP_15	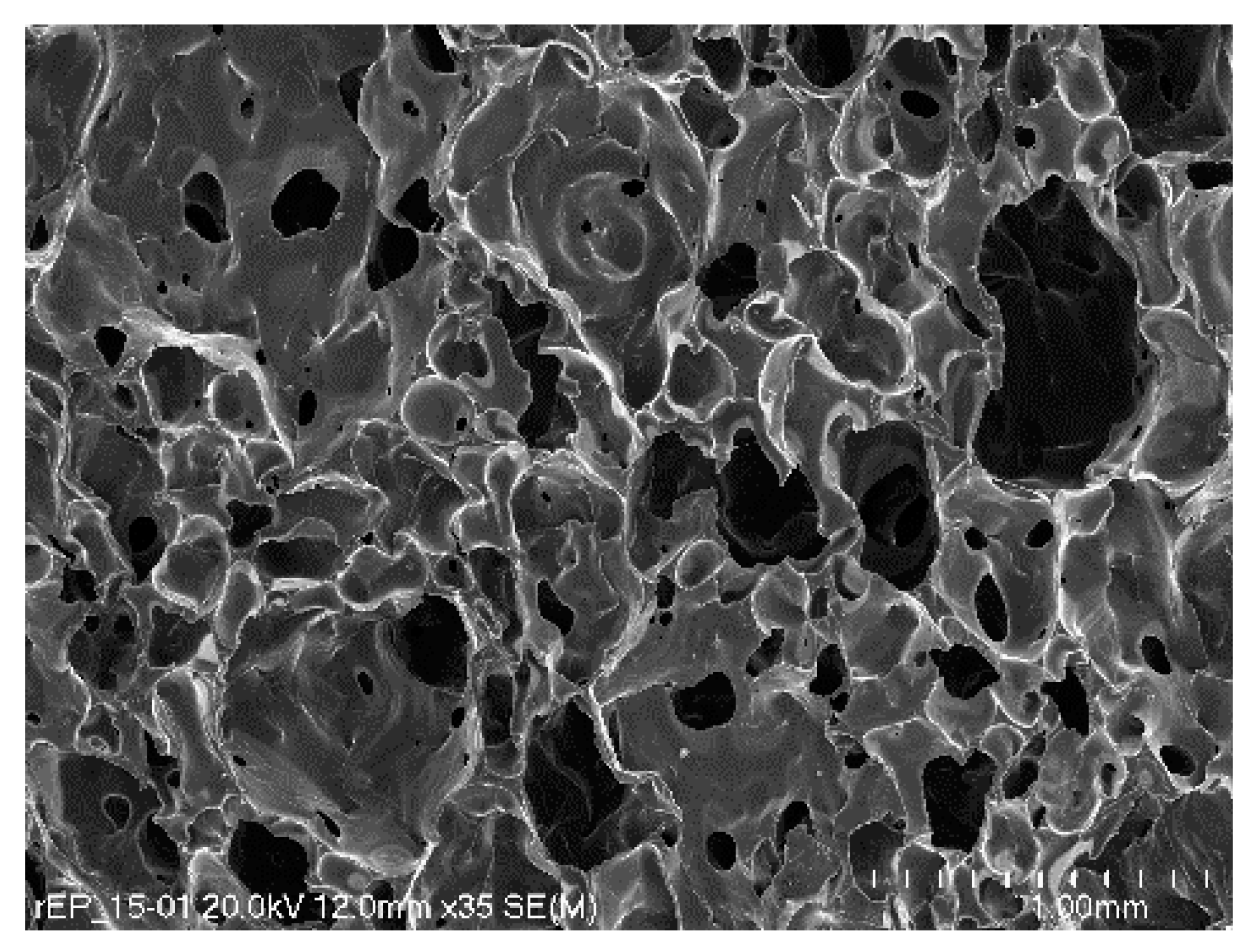	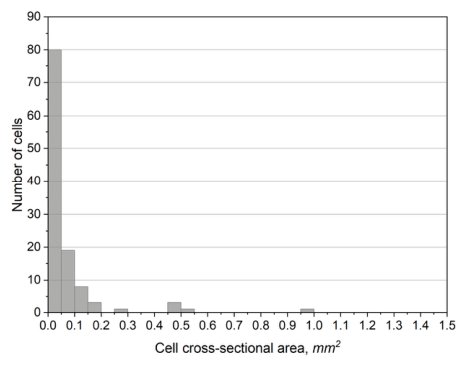

**Table 12:** SEM images and histograms of V_rFB cell areas.

Foam Symbol	SEM Photo	Histogram
V_rFB_0 (REF)	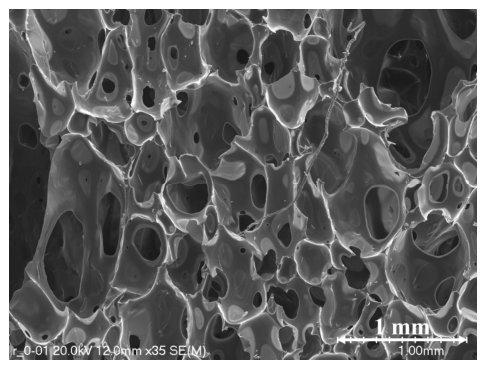	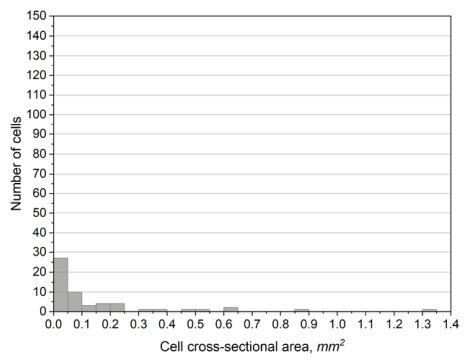
V_rFB_5	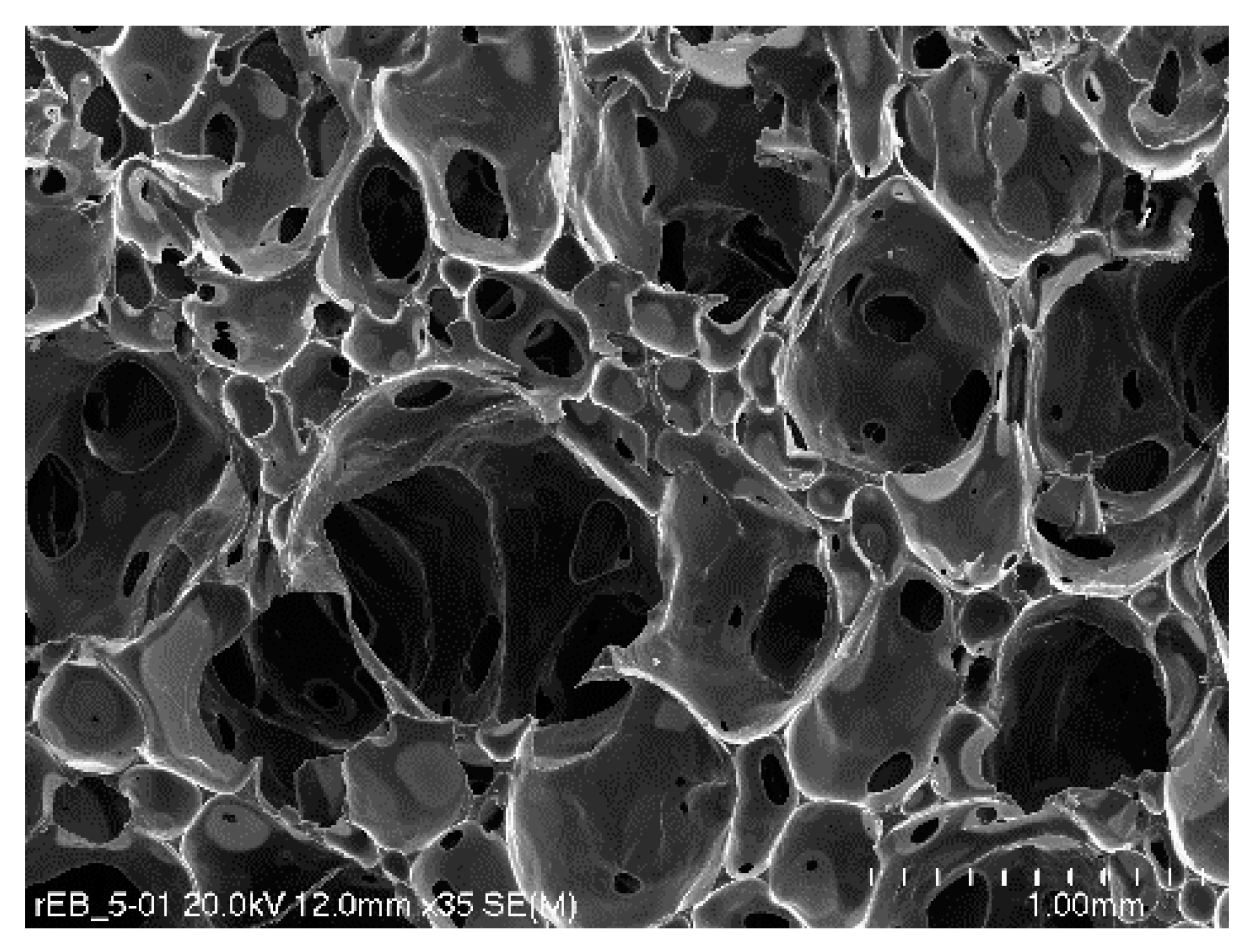	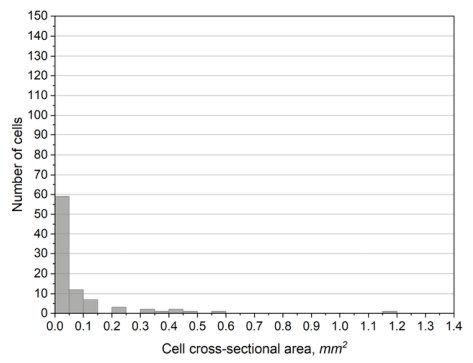
V_rFB_10	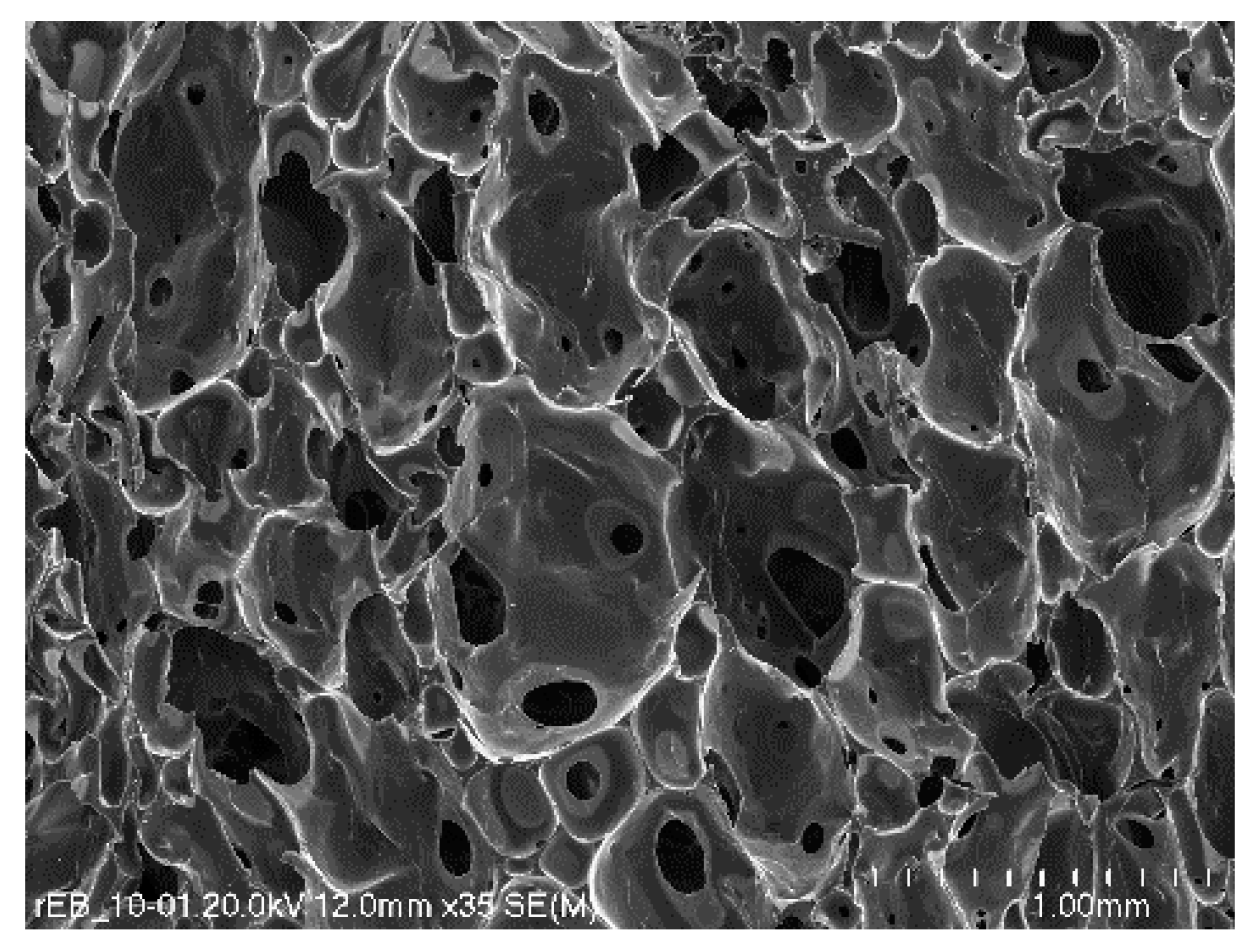	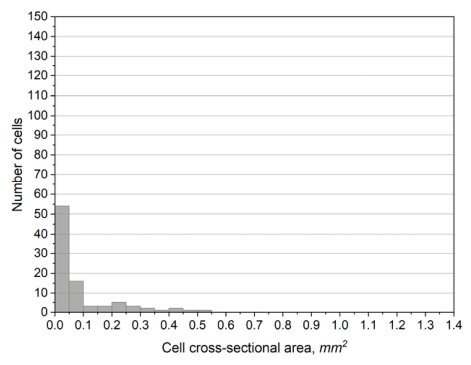
V_rFB_15	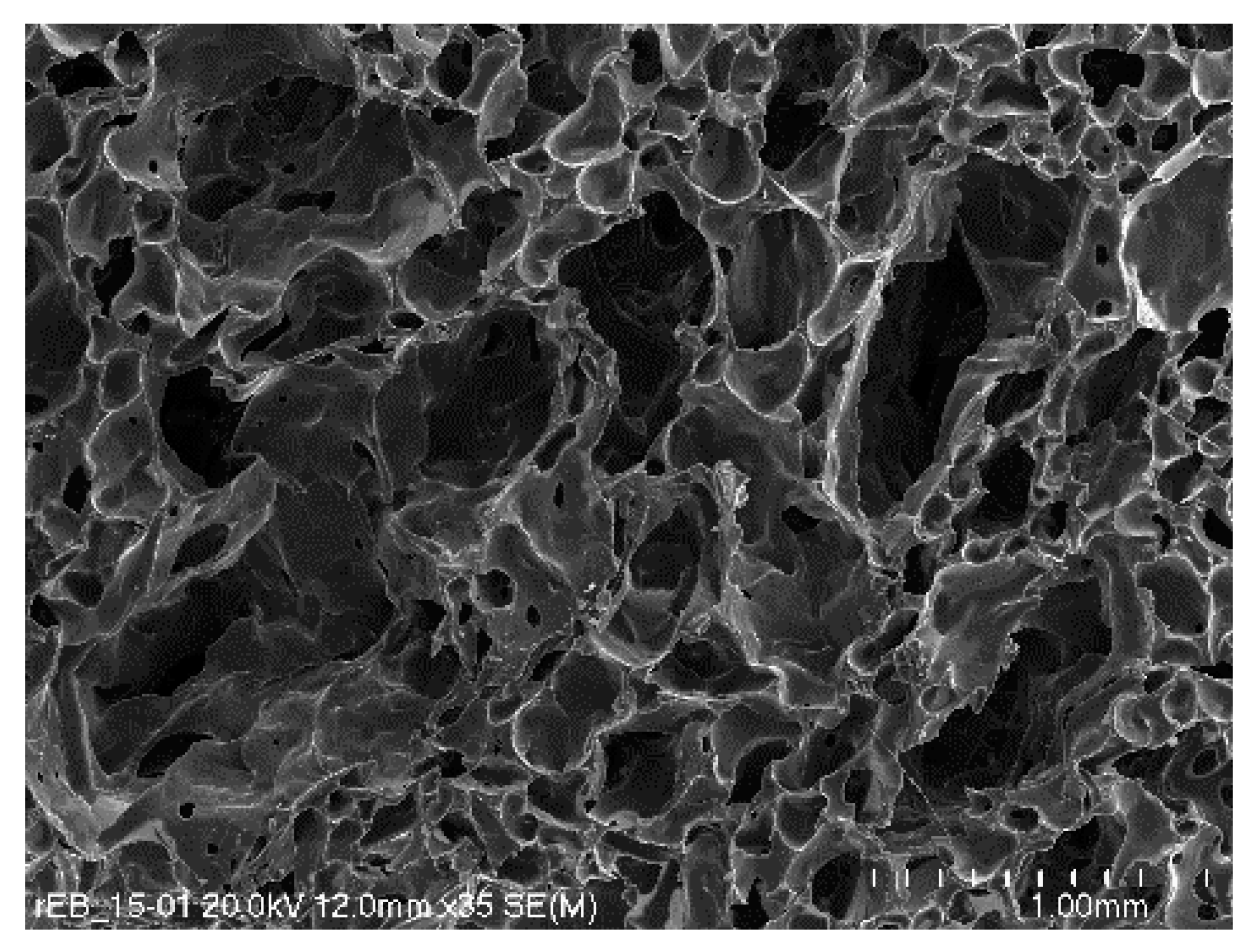	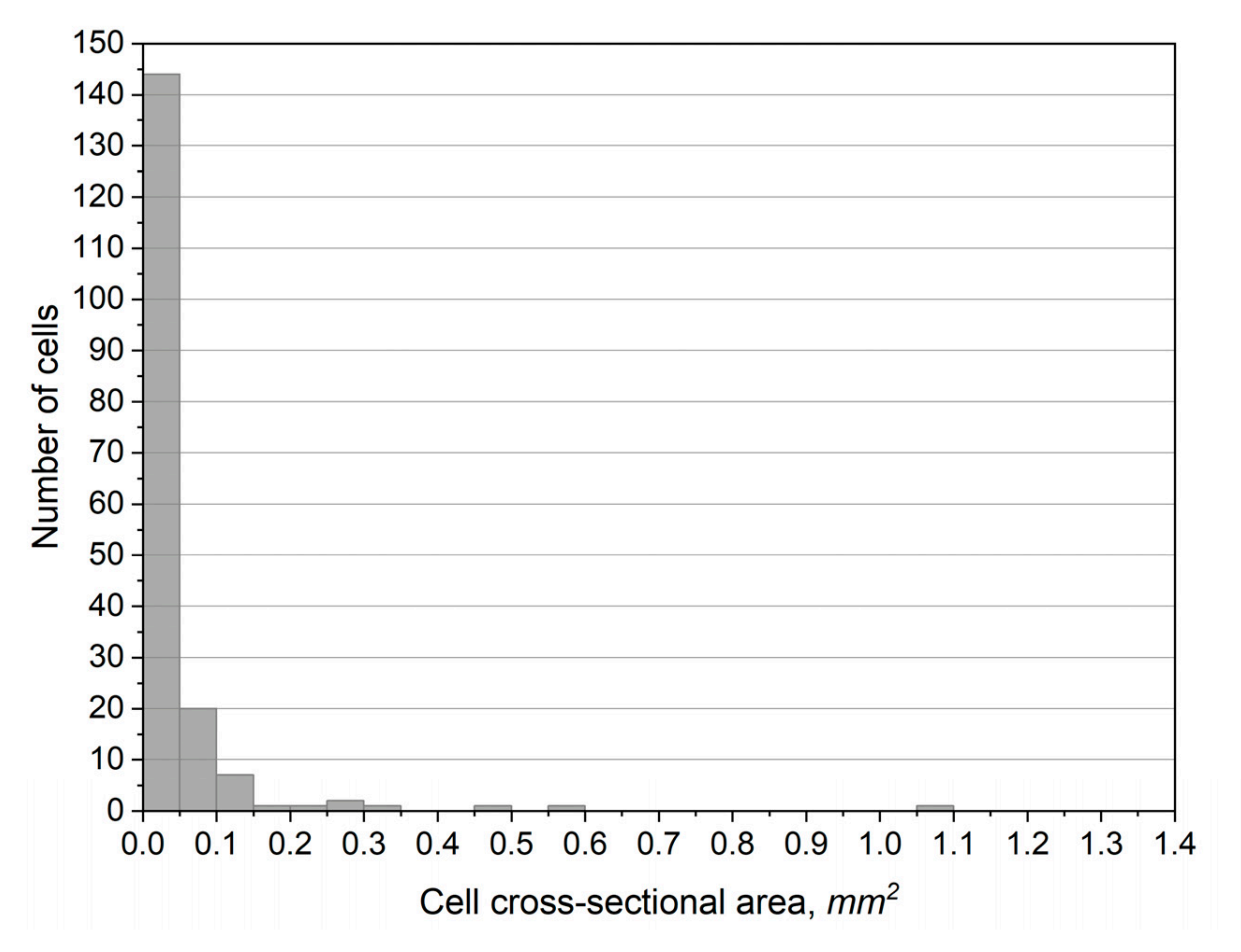

**Table 13:** Results of the analysis of the cellular structure of V_rFP and V_rFB.

Foam Symbol	Number of Cells per 1 mm^2^	Cell Cross-Sectional Area, mm^2^	Cell Anisotropy Coefficient	Cell Density, Cells/mm^3^
V_rFP_0/V_rFB_0 (REF)	18 ± 1.7	0.04 ± 0.005	1.3 ± 0.07	76 ± 10.8
V_rFP_5	6 ± 0.3	0.15 ± 0.020	1.1 ± 0.01	14 ± 1.0
V_rFP_10	7 ± 0.0	0.12 ± 0.001	1.2 ± 0.01	18 ± 0.0
V_rFP_15	12 ± 0.4	0.06 ± 0.003	1.2 ± 0.01	42 ± 2.2
V_rFB_5	10 ± 1.7	0.08 ± 0.008	1.1 ± 0.08	33 ± 8.2
V_rFB_10	9 ± 0.3	0.09 ± 0.003	1.3 ± 0.03	27 ± 1.3
V_rFB_15	10 ± 1.6	0.08 ± 0.013	1.0 ± 0.03	30 ± 7.3

**Table 14:** Content of flexible segments and rigid segments, and recovery time of V_rFP and V_rFB.

Foam Symbol	Flexible Segments, %	Rigid Segments, %	Recovery Time, s
V_rFP_0/V_rFB_0 (REF)	69.2	30.8	20 ± 0.0
V_rFP_5	68.0	32.0	31 ± 2.3
V_rFP_10	66.8	33.2	57 ± 6.1
V_rFP_15	65.6	34.4	>300
V_rFB_5	67.9	32.1	37 ± 2.3
V_rFB_10	66.5	33.5	93 ± 7.6
V_rFB_15	65.2	34.8	>300

## Data Availability

Dataset available on request from the authors.
